# PM2.5 as a driver of human health disorders: insights from the regulated cell death pathways

**DOI:** 10.1016/j.mmr.2026.100046

**Published:** 2026-06-18

**Authors:** Mohammad Amin Khalilzad, Seyyed Maysam Mousavi Shoar, Amirhosein Abbasi, Mina Shafiei Falavarjani, Mitra Khalilzad, Amirhossein Naseri, Sajad Najafi

**Affiliations:** a Department of Medical Biotechnology, National Institute of Genetic Engineering and Biotechnology, 14965-161, Tehran, Iran; b Department of Cell and Molecular Sciences, Faculty of Biological Sciences, Kharazmi University, 14911-15719, Tehran, Iran; c Brain Mapping Research Center, Shahid Beheshti University of Medical Sciences, 13336-35445, Tehran, Iran; d Department of Surgery, Taleghani Hospital, Shahid Beheshti University of Medical Sciences, 19857-11151, Tehran, Iran; e Department of Medical Biotechnology, School of Advanced Technologies in Medicine, Shahid Beheshti University of Medical Sciences, 19689-17313, Tehran, Iran; f Zoonotic Diseases Research Center, Ilam University of Medical Sciences, 69391-77143, Ilam, Iran; g Biotechnology and Medicinal Plants Research Center, Ilam University of Medical Sciences, 69391-77143, Ilam, Iran

**Keywords:** PM2.5, Air pollution, Regulated cell death (RCD), Ferroptosis, Oxidative stress

## Abstract

Fine particulate matter (PM2.5) is a prevalent environmental pollutant that has been well established as a contributor to morbidity on a global scale. An increasing body of scientific evidence suggests that PM2.5 promotes various modes of regulated cell death (RCD), including apoptosis, necroptosis, pyroptosis, PANoptosis, ferroptosis, and autophagy-dependent cell death, as well as potentially cuproptosis, across a broad spectrum of human diseases. These interconnected pathways demonstrate how exposure to pollutants induces oxidative stress, mitochondrial dysfunction, endoplasmic reticulum stress (ERS), and inflammatory responses. Alterations in lysosomal permeability serve as a critical link, connecting environmental pollutants to health conditions. Importantly, dysregulation of RCD mechanisms is associated with exposure to PM2.5 and numerous health disorders, such as cardiovascular diseases (CVDs), neurological conditions, respiratory illnesses, renal and hepatic dysfunctions, reproductive health issues, and ocular diseases. Collectively, RCD functions as a central molecular framework through which PM2.5 exposure accelerates the progression of pathological conditions. This review synthesizes recent mechanistic insights, identifies promising therapeutic candidates, and highlights critical knowledge gaps. It offers a strategic framework to guide future research endeavors aimed at mitigating the impact of PM2.5-induced RCD in human diseases. Additionally, we will identify and explore the research gaps that need to be addressed to effectively translate preclinical PM2.5 studies into clinical trials. We will also provide a comprehensive overview that both highlights these gaps and offers pathways to bridge them.

## Background

Particulate matter (PM) consists of microscopic solid and liquid particles suspended in the atmosphere, originating from a variety of sources, including diesel engine emissions, industrial manufacturing processes, construction activities, widespread wildfires, volcanic eruptions, and agricultural operations [1]. The physical characteristics of PM, such as particle size, shape, and density, influence both its residence time in the atmosphere and its contribution to air pollution. PM is classified according to aerodynamic diameter into three primary categories: PM0.1 (ultrafine particles smaller than 0.1 μm capable of penetrating deep into the pulmonary alveoli and entering the bloodstream), PM2.5 (fine particles less than 2.5 μm that reach the alveolar regions of the lungs), and PM10 (coarse particles smaller than 10 μm that typically deposit in the upper respiratory tract) [2]. PM0.1 and PM2.5 are of particular concern due to their ability to bypass the body’s natural defenses and enter systemic circulation, potentially causing inflammation and damage to vital organs [3]. PM2.5 accounts for the majority of the PM measured in routine air quality monitoring efforts [3]. Recent assessments from the Global Burden of Disease 2021 reveal a startling impact of air pollution; approximately 7.83 million lives were lost globally in 2021 due to long-term exposure to ambient PM2.5. This alarming data corresponds to an age-standardized mortality rate of about 95.69 deaths per 100,000 people [4].

A substantial body of mechanistic research suggests that PM2.5 primarily induces disease by disrupting regulated cell death (RCD) pathways [4], [5]. These programmed processes include apoptosis (a form of programmed cell death involved in the development and tissue homeostasis), ferroptosis (iron-dependent lipid peroxidation-driven cell death), pyroptosis (inflammatory cell death mediated by gasdermin pore formation), necroptosis [regulated necrosis involving receptor-interacting protein kinases (RIPK)], PANoptosis (a hybrid form combining features of pyroptosis, apoptosis, and necroptosis), and autophagy-related (ATG) cell death (a cellular degradation pathway that can result in cell death under stress conditions) [6]. These mechanisms enable cells to adapt to environmental or internal stressors or undergo controlled death to preserve tissue integrity. Unlike spontaneous necrosis, which is unregulated and often deleterious, these pathways respond to specific cellular cues in a regulated, programmed manner [6], [7]. PM2.5 influences various systems governing RCD, including iron management via ferritin and transferrin receptors [6], [8], redox homeostasis through reactive oxygen species (ROS) generation [9], mitochondrial integrity and function [10], lysosomal stability [6], [11], and endoplasmic reticulum (ER) signaling pathways [12]. The biochemical stress elicited by PM2.5 activates different forms of RCD, each exerting distinct effects on inflammation, metabolic processes, and cellular architecture [6], [13]. Understanding PM2.5 toxicity through this detailed framework has shifted perspectives, framing it as a multifaceted mechanism that directly precipitates cell death rather than solely causing organ-wide injury or dysfunction.

Collectively, RCD functions as a vital molecular framework that mediates the biological effects of PM2.5 exposure, thereby accelerating the development of an array of pathological conditions. These encompass cardiovascular diseases (CVDs), such as myocardial injury [14] and cardiac fibrosis [15]_;_ neurological disorders, including neurodegeneration [16], ischemic stroke, and epilepsy [17]; respiratory illnesses such as pulmonary fibrosis [18] and other immunological respiratory conditions [19]; reproductive health issues, including infertility and hormonal imbalances [6], [20]; renal and hepatic dysfunctions characterized by impaired filtration [21], [22]; and ocular diseases such as dry eye syndrome [23]. Targeting various aspects of RCD pathways has unlocked new, effective therapeutic strategies for these complex conditions. For example, ferroptosis inhibitors, such as ferrostatin-1 (Fer-1), are highly protective for the heart by preventing iron-dependent lipid peroxidation in cardiac tissues [24]. Additionally, emerging research indicates that exposure to PM2.5 is strongly linked to RCD-regulated cancers, such as lung cancer [25], through mechanisms involving oxidative stress and inflammation. While standard cancer treatments include chemotherapy and immunotherapy [6], [26], integrating targeted modulation of the RCD pathway will undoubtedly enhance treatment efficacy and overcome resistance, making it a critical focus for future research.

**Fig. 1:**
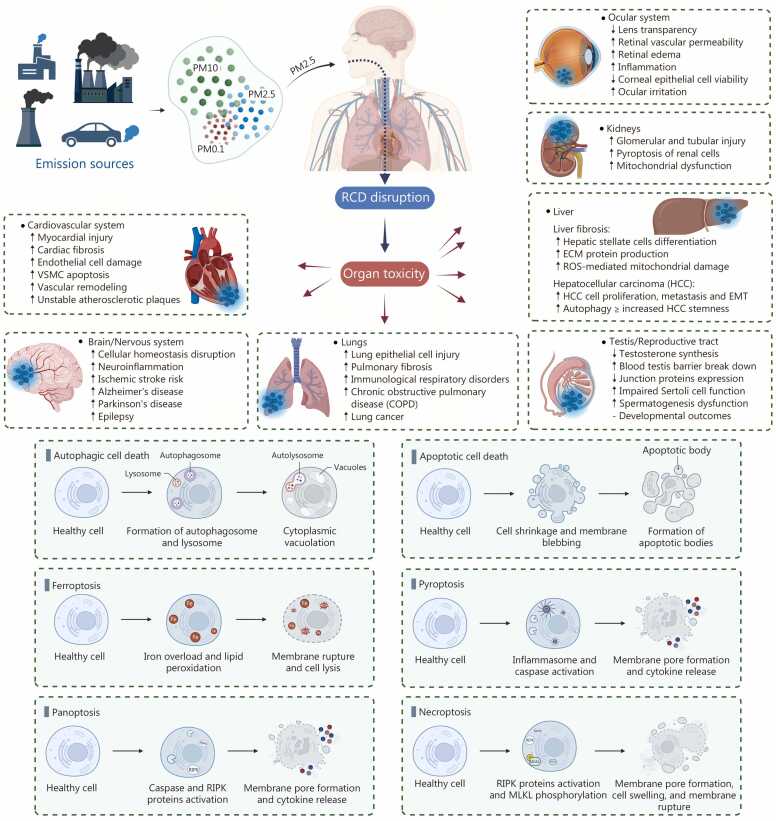
PM2.5-induced multi-organ toxicity and related regulated cell death pathways. PM2.5 significantly contributes to multisystem toxicity by inducing oxidative stress, inflammation, and cellular damage across multiple organs. Its harmful effects are primarily due to disruption of various regulated cell death pathways, including apoptosis (programmed cell death), ferroptosis (iron-dependent lipid peroxidation), pyroptosis (inflammatory cell death), autophagy (cellular degradation and recycling), necroptosis (programmed necrosis), and PANoptosis (a form of inflammatory cell death involving multiple pathways). By interfering with these processes, PM2.5 can lead to diverse disease conditions affecting the cardiovascular system (such as myocardial injury, cardiac fibrosis, vascular cell damage), the brain (such as contributing to neurodegenerative disorders, ischemic stroke, and epilepsy), lungs [lung epithelial cell injury, pulmonary fibrosis, immunological respiratory disorders, chronic obstructive pulmonary disease (COPD) and lung cancer], reproductive tract (impacting fertility), kidneys (causing glomerular and tubular injury), liver (liver fibrosis and hepatocellular carcinoma), and ocular system (leading to eye inflammation and damage). Created with BioRender.com. PM. Particulate matter; EMT. Epithelial-mesenchymal transition; RCD. Regulated cell death; ECM. Extracellular matrix; VSMC. Vascular smooth muscle cell; ROS. Reactive oxygen species; RIPK. Receptor-interacting protein kinase; MLKL. Mixed lineage kinase domain-like protein.

This review synthesizes the latest molecular insights into the mechanisms by which PM2.5 influences RCD pathways in critical organ systems. Organized around cell-death biology rather than specific disease outcomes, the discussion clarifies how PM2.5 affects cellular stress responses such as oxidative stress, mitochondrial dysfunction, and inflammasome activation that contribute to a range of diseases, such as chronic obstructive pulmonary disease (COPD), CVD, and renal failure **(**Fig. 1**)**. The review integrates current scientific findings, identifies research gaps, and highlights novel molecular targets, including specific signaling pathways and genetic markers. It also delineates key actions necessary for progress, such as targeted reductions in ambient PM2.5 levels, the development of biomarkers, and personalized therapeutic strategies. This biologically based, mechanistic approach aims to foster the development of effective interventions to mitigate the adverse health effects associated with PM2.5 exposure, particularly in the context of environmental and occupational factors.

## Regulated cell death

Extreme microenvironmental conditions, such as elevated pressure, shear forces, and temperature fluctuations, can lead to accidental cell death through the rapid degradation of cellular components [27]. In contrast, RCD results from milder homeostatic disturbances or developmental signals and may be influenced by pharmacological agents or genetic modifications. Furthermore, RCD can be initiated by external factors when adaptive mechanisms, such as autophagy, become ineffective [27]. This section will explore the various types of RCD in greater detail. Cellular death occurs at a staggering rate of millions per second in the human body, representing a common physiological process. Of the various forms of cell death, apoptosis is the most prevalent; however, other mechanisms, such as necroptosis, ferroptosis, pyroptosis, and autophagy, can also be induced by specific diseases [28]. In the third section of this review (PM2.5 and its contribution to various health disorders via regulated cell death), the detrimental effects of PM2.5 on the pathophysiology of various diseases are discussed. This segment emphasizes the influence of PM2.5 on key RCD mechanisms and how these processes contribute to disease progression. To maintain focus and avoid redundancy, an initial exploration of the fundamental mechanisms of RCD will be conducted, providing a comprehensive overview of their roles in health and disease. This foundational discussion will serve as a basis for the subsequent analysis of the impact of PM2.5.

### Apoptosis

**Fig. 2:**
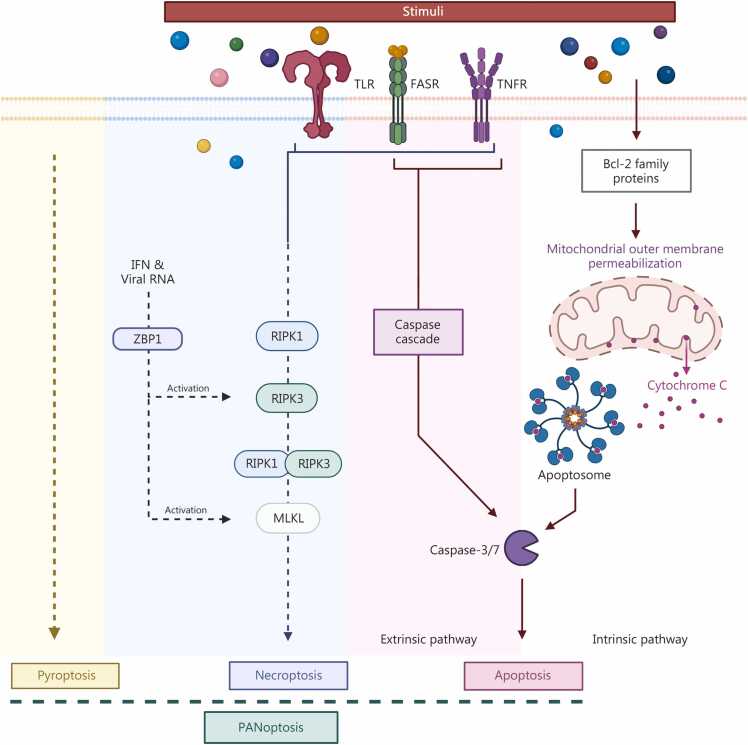
Schematic representation of the molecular pathways, with emphasis on the convergence and crosstalk among pyroptosis, necroptosis, and apoptosis, collectively framed as PANoptosis. The diagram highlights the involvement of pattern recognition receptors such as TLR, FASR, and TNFR in mediating distinct cell death pathways. Apoptosis is divided into extrinsic and intrinsic pathways: the extrinsic pathway involves death receptor-mediated activation of the caspase cascade (caspase-3/7), whereas the intrinsic pathway involves mitochondrial outer membrane permeabilization mediated by Bcl-2 family proteins, subsequent cytochrome C release, and apoptosome assembly. Necroptosis involves sequential activation of RIPK1, RIPK3, and MLKL downstream of death receptor stimulation. Pyroptosis is depicted as being triggered through IFN and viral RNA signaling via the ZBP1-RIPK3 axis. The integrative illustration delineates the mechanistic interplay between these regulated cell death modalities in response to stimuli, annotating critical signaling intermediates and major steps for scientific clarity and reproducibility. Created with BioRender.com. TLR. Toll-like receptor; FASR. Fas receptor; TNFR. Tumor necrosis factor receptor; IFN. Interferon; ZBP1. Z-DNA binding protein 1; RIPK1/3. Receptor-interacting protein kinase 1/3; MLKL. Mixed lineage kinase domain-like protein; Bcl-2. B-cell lymphoma-2.

Adults lose over 50 billion cells per day, highlighting the importance of understanding apoptosis as a key anti-inflammatory process in managing inflammation [29]. The immune system function, tissue preservation, and embryonic development depend on apoptosis [30]. Both internal and extrinsic signaling mechanisms bring on apoptosis. At the same time, the intrinsic pathway is initiated by cellular stress, which increases mitochondrial membrane permeability and releases proteins, such as cytochrome C, that are regulated by the B-cell lymphoma-2 (Bcl-2) family. The extrinsic pathway is activated when death ligands, such as tumor necrosis factor-α (TNF-α) and Fas, bind to their respective receptors **(**Fig. 2**)**. Apoptosis disruption is linked to cardiovascular problems, neurological illnesses, autoimmune diseases, and cancer [30]. Notably, the protease family known as caspases is essential for regulating apoptosis. They are classified into apoptotic and inflammatory caspases: apoptotic caspases control the programmed destruction of damaged cells, whereas inflammatory caspases induce pyroptosis and stimulate inflammation. Caspases are triggered by internal and extrinsic pathways, resulting in DNA fragmentation and cell death. Effective immune responses depend on the balance of various caspase types, highlighting their function in preventing illness and promoting health [31].

### Necroptosis

Necroptosis is a regulated form of cell death that differs from necrosis and apoptosis. It is initiated by death receptors, such as TNF receptor (TNFR) 1, TNFR2, and Fas receptor (FASR). When apoptosis is inhibited, the activation of three key proteins [RIPK1, RIPK3, and mixed lineage kinase domain-like protein (MLKL)] triggers the necroptotic process **(**Fig. 2**)**. This response leads to inflammation, organelle hypertrophy, and cell membrane rupture [32]. Over the past decade, necroptosis has gained recognition for its role in various biological processes [32]. Although distinct from necrosis, necroptosis shares similarities, including plasma membrane disruption and the subsequent release of intracellular contents [33]. Necroptosis is induced when proteins from the TNFR family are activated in the absence of caspase 8 activity, leading to the recruitment of RIPK1 and RIPK3. Activation of toll-like receptors (TLRs) can initiate necroptosis in either a RIPK1-dependent or RIPK1-independent manner. Intracellular Z-form DNA (Z-DNA) and RNA produced during viral replication can activate RIPK3 via Z-DNA binding protein 1 (ZBP1) [33]. Furthermore, interferon (IFN) induces activation of RIPK3 and MLKL through ZBP1, facilitating the formation of the RIPK1-RIPK3 complex **(**Fig. 2**)**. This mechanism is linked to several medical conditions, including acute kidney injury, inflammation, cancer, neurodegeneration, infectious diseases, cardiovascular issues, and dermatological disorders [27].

### Pyroptosis

**Fig. 3:**
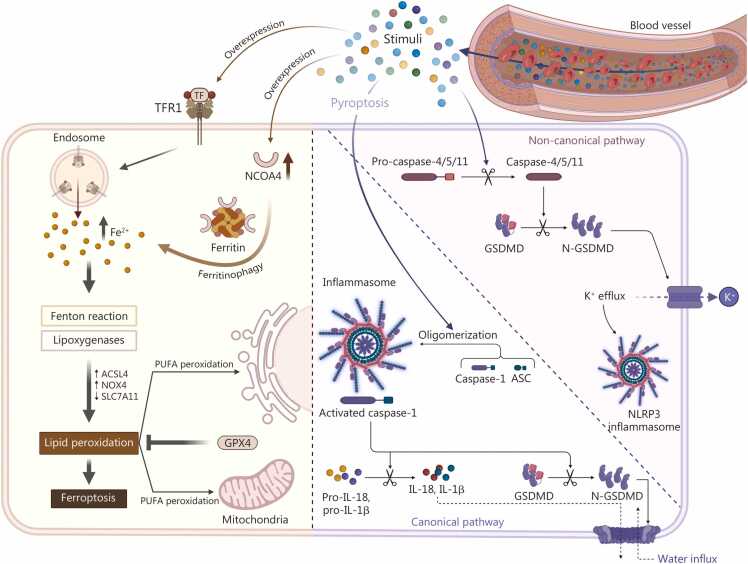
Mechanistic overview of pyroptosis and iron-dependent ferroptosis induced by stimulus exposure. Pyroptosis pathways (right) are activated by PM2.5, involving both canonical and non-canonical inflammasome signaling. The canonical pathway features inflammasome assembly with activated caspase-1 cleaving proinflammatory cytokines IL-18 and IL-1β, and GSDMD to its active N-terminal fragment (N-GSDMD), promoting membrane pore formation and cell lysis. The non-canonical pathway involves caspases-4/5/11 cleaving GSDMD directly. Both pathways culminate in potassium efflux and water influx, characteristic of pyroptotic cell death. Ferroptosis (left) is initiated by the overexpression of TFR1 and NCOA4, leading to ferritinophagy and increased intracellular Fe^2+^ release. Elevated iron catalyzes ROS production via the Fenton reaction and lipoxygenase activity, triggering lipid peroxidation and ferroptotic cell death. Key regulatory molecules include ACSL4, NOX4, and SLC7A11, with GPX4 acting to inhibit lipid peroxidation. Created with BioRender.com. PUFA. Polyunsaturated fatty acid; ASC. Apoptosis-associated speck-like protein containing a CARD; TF. Transferrin; TFR1. Transferrin receptor 1; NCOA4. Nuclear receptor coactivator 4; ACSL4. Acyl-CoA synthetase long-chain family member 4; NOX4. NADPH oxidase 4; SLC7A11. Solute carrier family 7 member 11; GPX4. Glutathione peroxidase 4; N-GSDMD. N-terminal fragment gasdermin D; IL. Interleukin; NLRP3. NLR family pyrin domain-containing 3; ROS. Reactive oxygen species.

Pyroptosis derives its name from the combination of “pyro”, which indicates fire, reflecting the inflammatory characteristics associated with this process, and “ptosis”, meaning falling, which aligns with other forms of RCD [34]. Pyroptosis is a form of RCD that produces cytokines and granzymes, which subsequently contribute to the activation of the apoptosis pathway **(**Fig. 3**)**. This process can be induced by pathogens, chemotherapeutic agents, and PM2.5 and is characterized by inflammation, DNA damage, and chromatin condensation [34], [35]. Unlike apoptosis, during pyroptosis, the cytoplasm flattens due to the leakage of the plasma membrane [36]. This phenomenon is facilitated by the release of granzymes or the activation of caspases, instigating the oligomerization of gasdermin and the formation of pores in the plasma membrane. The influx of water through these pores leads to osmotic lysis and cellular swelling, ultimately resulting in membrane rupture and the release of inflammatory cytokines interleukin (IL)-1β and IL-18 [37].

The cleavage of gasdermin D (GSDMD) and the release of IL-1β and IL-18 are closely associated with the assembly of inflammasomes, which mediate canonical pyroptotic death. Inflammasomes are activated when the host exhibits resistance to microbial infection, thus playing a crucial role in developing adaptive immune responses [37]. They are also implicated in various non-microbial diseases [34]. NOD-like receptor family pyrin domain containing (NLRP)1, NLRP3, NLR-family CARD domain-containing protein 4 (NLRC4), absent in melanoma 2 (AIM2), and pyrin are the primary parts of canonical inflammasomes, which are assembled by the inflammasome sensors. These elements consist of leucine-rich repeat-containing proteins known as NOD-like receptors, pro-caspase-1, and the adaptor protein apoptosis-associated speck-like protein containing a caspase recruitment domain (ASC) [38].

### PANoptosis

In 2019, Malireddi *et al*. [39] discovered a novel kind of cell death called PANoptosis. Though it differs from each of them, it shares characteristics with apoptosis, necroptosis, and pyroptosis **(**Fig. 2**)**. In reaction to pathogenic components, a complex network of receptors and signaling pathways forms the PANoptosome complex, which in turn causes cell death [37], [40]. It has emerged as a major area of study in illnesses, including cancer, immunological problems, and infections.

### Ferroptosis

Ferroptosis, introduced in 2012, refers to cell death linked to small-molecule drugs that inhibit rat sarcoma-mutant cancer cell growth [37], [41]. It emerged from studies on cancer cell death due to cysteine depletion and the concept of oxytosis, which is neuron death caused by glutamate toxicity [42]_._ Ferroptosis involves inflammatory cell death, marked by lipid oxidation products and damage-associated molecular patterns, such as DNA and high mobility group box 1. The process involves necrosis-like changes, including chromatin condensation, cytoplasmic and organelle enlargement, and loss of plasma membrane integrity. It can spread to adjacent cells, causing rounding, detachment, and increased autophagosome formation [43]. Ferroptotic cells also display abnormal mitochondrial architecture [43]. This unique form of cell death is characterized by iron-dependent lipid peroxidation **(**Fig. 3**)**. Several metabolic pathways influence it, including redox balance, iron metabolism, and the metabolism of amino acids, lipids, and carbohydrates [44]. Ferroptosis has been implicated in various organ damage and degenerative disorders while playing significant roles in CVD, liver disease, and respiratory disease [44], [45]. To date, a total of 264 driving targets and 238 inhibition targets have been identified for the modification of ferroptosis [46]. Nuclear receptor coactivator 4 (NCOA4), nicotinamide adenine dinucleotide phosphate (NADPH) oxidase 4, and Kelch-like ECH-associated protein 1 (KEAP1) are important targets for the induction of ferroptosis [46] and, accordingly, many ferroptosis inducers like erastin and rat sarcoma synthetic lethal 3 have been introduced for the promotion of ferroptotic cell death [47]. On the other side, ferritin heavy chain 1 (FTH1), solute carrier family 7 member 11 (SLC7A11), glutathione (GSH) peroxidase (GPX) 4, ferroptosis suppressor protein 1, and nuclear factor erythroid 2-related factor 2 (NRF2) are described as well-known targets for suppressing ferroptosis [46], which along with other ferroptosis players can be targeted by various compounds [48]. The regulation of cell iron levels depends on proteins including ferritin, transferrin, hepcidin, ferroportin, ferroportin 1, and transferrin receptor 1 [49]. Nonetheless, a compromise in cellular defenses can lead to polyunsaturated fatty acid peroxidation, facilitating ferroptosis [46].

### Autophagy

**Fig. 4:**
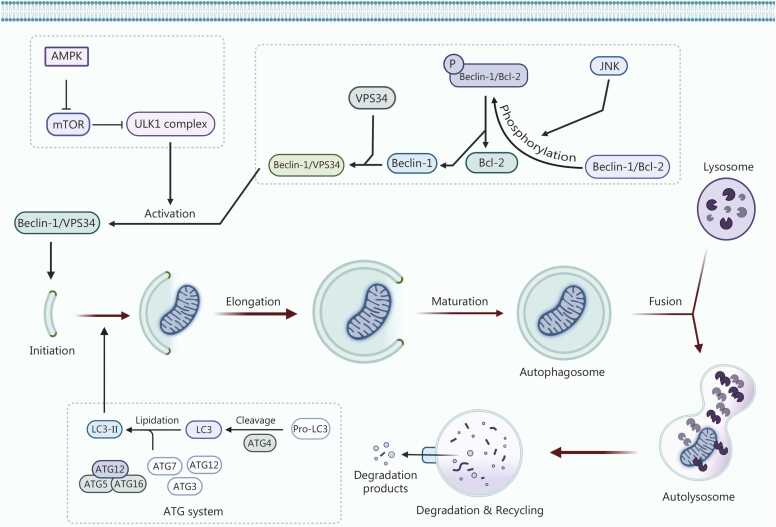
Schematic illustration of the mechanism and regulatory pathways governing macroautophagy. Autophagy initiation is controlled by AMPK-mediated inhibition of mTOR, leading to activation of the ULK1 complex, which subsequently activates the Beclin-1/VPS34 complex essential for autophagosome nucleation. The Beclin-1/VPS34 complex activity is regulated by interactions with Beclin-1 and Bcl-2, modulated via phosphorylation by JNK, which disrupts the Beclin-1/Bcl-2 complex, promoting autophagy. Autophagic vesicle elongation, maturation, and autophagosome formation involve the conjugation and lipidation of LC3 to LC3-II, facilitated by ATG proteins in the ATG system, including ATG3, ATG4, ATG5, ATG7, ATG12, and ATG16. The mature autophagosome fuses with lysosomes to form the autolysosome, where degradation and recycling of cellular components occur, aiding in cellular homeostasis. This figure integrates key molecular players and phases in macroautophagy, highlighting its tightly regulated nature and essential role in cellular quality control mechanisms. Created with BioRender.com. AMPK. AMP-activated protein kinase; mTOR. Mammalian target of rapamycin; ULK1. Unc-51-like autophagy activating kinase 1; VPS34. Vacuolar protein sorting 34; JNK. c-Jun N-terminal kinase; Bcl-2. B-cell lymphoma-2; LC3-II. Microtubule-associated protein 1 A/1B-light chain 3-II; ATG. Autophagy-related.

Autophagy is the process by which cellular constituents, such as proteins and organelles, are delivered to the lysosome or vacuole for breakdown [50]. Microautophagy and macroautophagy are the two main autophagy systems seen in eukaryotic cells [50], [51]. In the process of macroautophagy, a double-membraned structure referred to as the autophagosome is generated when a phagophore, characterized by its cup-shaped membrane structure, emerges near the ER, elongates, bends, and subsequently closes via membrane fission. Upon fusion with lysosomes, the contents of the autophagosome are broken down [50], [52] **(**Fig. 4**)**. In contrast, the endosomal or lysosomal membrane invaginates during microautophagy, directly engulfing a piece of cytoplasm [53]. The primary physiological roles of macroautophagy and microautophagy are the selective destruction of proteins, organelles, and foreign materials to maintain cellular homeostasis and provide nutrients as needed [50], [54]. The intricate self-degradation process known as autophagy involves several key components, including the Beclin-1/vacuolar protein sorting 34 (VPS34) complex, c-Jun N-terminal kinase (JNK), ATG proteins, microtubule-associated protein 1 A/1B-light chain 3 (LC3)-I and -II, and various signaling pathways [55].

The initiation of autophagy is regulated by specific signaling pathways, wherein adenosine monophosphate-activated protein kinase (AMPK) inhibits the formation of the mechanistic target of rapamycin complex I (mTORC1) complex. The ATG5-ATG12 complex interacts with ATG16, forming a polymer complex that fuses with autophagic vesicles. Moreover, the Beclin-1/VPS34 complex prolongs the survival of autophagic vesicles, while JNK promotes the breakdown of the Beclin-1/Bcl-2 and Bcl-2-interacting mediator of cell death complexes. LC3 is incorporated by autophagosomes, and autolysosomes are created when lysosomes and autophagosomes combine [56].

### Cell death-associated cellular organelles mitochondria

Mitophagy, the mechanism responsible for removing outer mitochondrial membrane proteins, facilitates the upregulation of antiapoptotic proteins, thereby enhancing cellular survival. This process is primarily employed to eliminate damaged mitochondria, contributing to the prevention of apoptosis [56], [57]. However, an excessive occurrence of mitophagy may inadvertently trigger apoptosis by degrading functional mitochondria, disrupting mitochondrial homeostasis, and ultimately promoting cell death [56], [58]. Furthermore, mitophagy is instrumental in regulating pyroptosis, notably through pathways mediated by the NLRP3 inflammasome. Moderate levels of mitophagy can effectively prevent pyroptosis by removing damaged mitochondria and reducing mitochondrial ROS (mtROS) generation [59]. In contrast, mtROS are significant contributors to necroptosis, as they promote the autophosphorylation of RIPK1, which subsequently recruits RIPK3, culminating in the formation of the necrosome [60]. Thus, the clearance of mtROS via mitophagy may serve to inhibit necroptosis [60]; however, a previous study reports contradictory findings, indicating that extensive depletion of mitochondria via mitophagy does not compromise necroptosis [60], [61]. Numerous studies also indicate that mitophagy has a positive regulatory effect on ferroptosis [62], [63]. The accumulation of cellular ROS is crucial in this process. Generally, mitophagy is known to reduce mtROS, which represents a primary source of cellular ROS [60], [62]. Nonetheless, one study highlighted that while mitophagy decreased mtROS levels, it concurrently led to an increase in overall cellular ROS, thus promoting ferroptosis. This observation suggests that additional pathways are involved in elevating cellular ROS levels during mitophagy-induced ferroptosis [64].

### Endoplasmic reticulum

Cell survival depends on proteostasis, and illnesses, such as metabolic, neurological, oncological, and cardiovascular conditions, may result from an imbalance in this process. The ER, which serves as a quality-control organelle, ensures that only correctly folded proteins are transported from ER vesicles. ATPases, chaperones, and systems, including the immunoglobulin-binding protein, glucose-regulated protein 94, the ubiquitin-proteasome system, and the lysosome-autophagy pathways, are all involved in this quality control [65]. ER-associated degradation is the process by which proteins that misfold under stress are taken out of the folding machinery and transferred to the cytosol to be broken down by the ubiquitin-proteasome system [66], [67]. The unfolded protein response (UPR) is brought on by ER stress (ERS), which is caused by persistently misfolded proteins [68]. UPR can modify gene expression, stop protein synthesis, and control cell destiny [69]. Negative external stimuli cause ERS, which may result in autophagy, apoptosis, ferroptosis, and pyroptosis, among other types of cell death [70]. Additionally, ER-associated degradation, protein synthesis, autophagy, oxidative stress, mitochondrial malfunction, and metabolism are all impacted by ERS [68].

## PM2.5 and its contribution to various health disorders via regulated cell death

PM2.5 plays a significant role in various health disorders. Recent evidence has increasingly underscored the contribution of PM2.5 alongside RCD in this context. The following section provides a comprehensive discussion on PM2.5 and its implications for multiple health disorders, particularly through the mechanisms associated with RCD.

### Cardiovascular diseases

The risk of CVD increases with age. In the context of CVD, PM2.5 plays a significant role through the implementation of various RCD mechanisms. Numerous cellular organelles, including mitochondria and the ER, may influence the mechanisms through which PM2.5 exerts its effects. This section will investigate the various connections between PM2.5 and CVD, focusing on the perspectives provided by RCD mechanisms.

#### PM2.5 and cardiomyocytes

One significant factor contributing to the cardiovascular side effects associated with PM2.5 is its pro-apoptotic effect on cardiomyocytes. However, the molecular mechanisms underlying this interaction remain inadequately understood [68], [71]. Researchers used human cardiomyocytes (AC16) to measure the cardiac damage associated with PM2.5 exposure. They observed a rise in lactate dehydrogenase (LDH) release and a dose-dependent decline in cell viability. Additionally, exposure to PM2.5 was associated with increased generation of ROS and malondialdehyde (MDA) and decreased levels of GPX4 and superoxide dismutase (SOD). Measurements of the mitochondrial membrane potential (MMP) and ultrastructural analysis confirmed the presence of mitochondrial damage. Interestingly, the apoptotic rate in AC16 cells was markedly increased by PM2.5 exposure. Furthermore, in the mitochondria-mediated apoptosis pathway, the anti-apoptotic protein Bcl-2 was reduced, while the levels of caspase-3, caspase-9, and Bcl-2-associated X protein (Bax) were elevated [72].

Researchers evaluated the cardiotoxic effects of traffic-related PM2.5 (TRPM2.5) in a different investigation employing AC16. The findings indicate that exposure to TRPM2.5 for 24 h initiated autophagy, apoptosis, and ERS. Sestrin2 was identified as a crucial mediator in the signaling pathways associated with apoptosis and autophagy [73]. The downregulation of protein kinase RNA-like endoplasmic reticulum kinase (PERK) in AC16 cells exposed to TRPM2.5 lowers the expression of proteins involved in autophagy, apoptosis, and ERS. According to this finding, ERS could trigger apoptosis and autophagy via the PERK/Sestrin2 signaling pathway. Furthermore, when 3-methyladenine was used to block autophagy, a drop in ATG markers and increased apoptosis were seen, indicating that TRPM2.5 exposure triggers protective autophagy [73]. This observation suggests that autophagy may serve a dual role in relation to PM2.5 exposure. While substantial evidence indicates the adverse effects of autophagy activation following PM2.5 exposure, it has now become apparent that autophagy may also exert a protective effect by modulating apoptosis.

The capacity of PM2.5 to promote cellular senescence in cardiomyocytes significantly contributes to its cardiovascular deleterious effects. Cellular senescence was seen in H9c2 cardiomyoblasts after exposure to extractable organic matter (EOM) from PM2.5. Higher p16 and p21 levels, more histone H3 lysine 9 trimethylation foci, and β-galactosidase-positive cells were all indicators of this. Exposure to EOM also raised intracellular and mtROS levels and enhanced DNA damage. The translocation of aryl hydrocarbon receptor (AhR) to the nucleus and the increase of cytochrome P450 1A1 (CYP1A1) and CYP1B1 expression demonstrated that EOM also activated the AhR pathway. Notably, oxidative damage and cellular senescence brought on by EOM were successfully decreased by an AhR antagonist [74].

#### Myocardial injury

Although the exact processes behind CVDs and PM2.5 are still unknown, recent research has shown a substantial correlation between exposure to PM2.5 and CVDs, especially in instances of hyperbetalipoproteinemia. For example, researchers utilized H9c2 cells and hyperlipidemic murine models to examine the impact of PM2.5 on myocardial injury [75]. Findings from high-fat animal models indicated that exposure to PM2.5 resulted in considerable cardiac damage, accompanied by oxidative stress and pyroptosis. Furthermore, the inhibition of pyroptosis using disulfiram reduced cardiac damage and pyroptosis levels, suggesting that PM2.5 activates the pyroptosis pathway, ultimately culminating in cell death and myocardial injury [75]. The well-established cardiotoxic consequences of PM2.5 exposure include a decreased heart’s capacity to withstand revascularization treatments and an increased vulnerability to ischemia-reperfusion injury [76]. The timing and processes behind this increased sensitivity are examined in a distinct study. Over the course of 7, 14, and 21 d, female Wistar rats were exposed to 250 μg/m^3^ of PM2.5 for 3 h every day. Significant heart damage, as shown by larger infarct sizes and higher injury biomarkers, as well as a decline in cardiac function, was seen by the 14th day of exposure. The adverse effects were linked to apoptosis and impaired mitochondrial functionality, which included diminished bioenergetics and compromised mitochondrial DNA quality control, along with inactivation of cardioprotective phosphoinositide 3-kinase (PI3K)/protein kinase B (Akt)/AMPK signaling pathways. Examination of cardiac tissue revealed an augmented accumulation of metals within the mitochondria. To assess the adverse effects of heavy metal ions on the cardiovascular system, ethylenediaminetetraacetic acid was employed as a chelating agent for metal ions in myocardial cell studies. Both rat myocardial cells and H9c2 cell lines demonstrated a significant reduction in the detrimental impacts of cardiac ischemia-reperfusion injury when metals associated with PM2.5 were chelated using ethylenediaminetetraacetic acid [77].

Copper, a heavy metal found in PM2.5 particles, plays a crucial role in cellular health [78], [79]. When it accumulates excessively in the mitochondria, it can trigger a distinctive form of cell death known as cuproptosis [78]. Interestingly, there is currently a significant research gap regarding the relationship between cuproptosis and PM2.5. This unexplored territory presents an exciting opportunity for investigation that could pave the way for innovative therapeutic strategies.

#### Cardiac fibrosis

To investigate the effects of PM2.5 on heart fibrosis through RCD, researchers employed both *in vitro* and *in vivo* experimental models. Their findings indicated that mice subjected to PM2.5 exposure exhibited the development of heart fibrosis. By upregulating the transcription factor Yin Yang 1 protein, the expression of NCOA4 was enhanced, thereby leading to the degradation of ferritin [24]. The Fenton reaction, associated with ferroptosis and lipid peroxidation, was initiated by the release of free iron due to this degradation. Notably, silencing NCOA4 protected against PM2.5-induced cell death by reducing ferroptosis and partially restoring FTH1 (also referred to as FHC) protein levels in HL-1 cells, which indicates a drop in iron accumulation. Additionally, using the ferroptosis inhibitor Fer-1 reduced the negative effects of ferritinophagy-mediated ferroptosis on heart fibrosis in mice exposed to PM2.5 [24].

The concept of cardiac remodeling holds significant relevance in the study of cardiac fibrosis [80]. Exposure to PM2.5 is significantly associated with cardiac remodeling, particularly cardiac hypertrophy. It is known that ferroptosis is a crucial process in this regard; however, it is yet unknown how exactly PM2.5 causes ventricular hypertrophy via ferroptosis. Li *et al*. [15] investigated the effects of PM2.5 on cardiac hypertrophy while exploring the potential beneficial role of mitoquinone (MitoQ). Their findings indicate that PM2.5 exposure leads to enlargement and dysfunction of cardiac tissue in mice, characterized by ferroptotic features, such as iron deficiency, lipid peroxidation, mitochondrial damage, and altered expression of essential biomolecules. Notably, treatment with MitoQ, which enhances the resistance of mitochondria to lipid peroxidation, effectively alleviated these symptoms. Further research demonstrated that administration of PM2.5 to AC16 cells, in conjunction with the ferroptosis activator erastin, triggered ferritinophagy, lipid peroxidation, mitochondrial dysfunction, and elevated levels of free iron. This process activated mitophagy, increasing free iron levels and heightened susceptibility of AC16 cells to ferroptosis. Additionally, Fer-1 mitigated iron overload and reduced PM2.5-induced cytotoxicity in the cytoplasm and mitochondria of AC16 cells. The results illustrate that dysregulated iron metabolism associated with ferroptosis due to PM2.5 initiates a cascade involving the activation of mitophagy and ferritinophagy. Moreover, *NCOA4* knockdown effectively rectified the iron dysregulation and lipid peroxidation induced by PM2.5, diminishing ferroptotic activity [15].

Approximately 90% of adenosine triphosphate (ATP) is produced by mitochondria, which make up about 30% of a cardiomyocyte’s volume [15], [81]. Thus, it is critical to comprehend how mitochondrial dysfunction and related cardiotoxic consequences are brought on by PM2.5 exposure. A recent study shed insight into the processes behind PM2.5-induced ventricular hypertrophy by examining calcium excess and mitochondrial dysfunction. Over the course of 4 weeks, male and female BALB/c mice were exposed to different levels of PM2.5 (1.28, 5.5, and 11 mg/kg body weight). In addition to notable changes in mitochondrial architecture, including rupture and dispersion, the high-dose group showed symptoms of myocardial edema and cardiac hypertrophy. A 24-hour exposure to PM2.5 caused the opening of mitochondrial permeability transition pores, which in turn decreased respiratory metabolism, decreased ATP synthesis, increased calcium levels, and diminished MMP, according to *in vitro* studies conducted using AC16 cells. Additionally, the use of a calcium chelator reduced the mitochondrial damage in cells treated with PM2.5. The results indicated that the excess calcium induced by PM2.5 exposure activates the mTOR/Akt/glycogen synthase kinase 3β (GSK-3β) signaling pathway, contributing to cardiac hypertrophy and mitochondrial dysfunction [82]. Research indicates that resident cardiac macrophages exhibiting proinflammatory polarization augment the cleavage of the receptor tyrosine kinase [myeloid-epithelial-reproductive receptor tyrosine kinase (MerTK)], which results in elevated levels of soluble MerTK (solMER) [83]. This pathway is implicated in myocardial damage and a decline in cardiac function due to its detrimental effect on cardiac healing processes [84]. An investigation was conducted to assess the impact of PM2.5 exposure on myocardial injury, left ventricular systolic performance, and solMER levels in otherwise healthy children. Plasma samples were analyzed for solMER and creatine kinase MB (CK-MB) levels, and each participant’s chronic daily intake (CDI) of PM2.5 was measured. The findings revealed that increased concentrations of solMER and CK-MB were associated with reduced stroke volume; notably, children in the exposed cohort exhibited higher CDI levels. Specifically, elevated solMER levels correlated with increased CDI. Furthermore, heightened CK-MB and diminished stroke volume were linked to increased solMER levels, and a positive correlation was identified between CDI and CK-MB mediated by solMER [83].

As mentioned, cardiac tissue fibrosis represents a significant challenge in CVD. Recent research indicates that the application of anti-fibrotic and anti-inflammatory biomaterials may present a novel avenue for addressing cardiac fibrosis [85]. In this context, placental tissue derivatives encompass diverse cellular and acellular components, demonstrating well-documented anti-fibrotic properties [86]. These properties are attributed to their ability to modulate the differentiation of myofibroblasts into fibroblasts and their anti-apoptotic effects on dermal tissue scars [87]. This understanding leads us to hypothesize that the implementation of such biomaterials in cardiac tissue could be a promising area of investigation for the treatment of PM2.5-induced cardiac fibrosis.

#### Vascular system, endothelial cells, and vascular smooth muscle cells

There is a connection between exposure to PM2.5 and ferroptosis of endothelial cells. However, the underlying processes of this connection are still not well understood. Wang *et al*. [88] examined intracellular iron levels, ROS production, lipid peroxidation, and certain ferroptosis markers to determine the role ferroptosis plays in endothelial cell damage caused by PM2.5. The results indicate that although GSH, GPX, and NADPH activities decrease with PM2.5 exposure, iron and ROS levels in cells rise. These alterations contribute to the facilitation of ferroptosis. Ferroptosis may also be influenced by reduced iron absorption and storage activities, as shown by the altered expression of iron-related proteins, such as transferrin receptor, FTH1, and ferritin light chain (FTL). Notably, by reestablishing antioxidant defense systems and improving iron metabolism, the iron chelator deferoxamine mesylate and the lipid peroxidation inhibitor Fer-1 have partially reversed these effects [88].

Excessive ERS serves as a critical precursor for both autophagy and apoptosis in human endothelial cells, and it has been established that the internalization of PM2.5 triggers this stress [89]. Utilizing 4-phenylbutyrate (4-PBA), an ERS inhibitor, effectively reduces apoptosis rates and decreases LC3-II expression, thereby supporting this assertion. Autophagy protects endothelial cells against PM2.5-induced cell death without influencing ERS. Furthermore, PM2.5 exposure reduces endothelial cell survival by suppressing autophagosome-lysosome fusion, which disrupts autophagic flow [89]. PM2.5 exposure has also been shown to reduce survival and proliferation of human umbilical vein endothelial cells (HUVECs) in a dose-dependent manner. Simultaneously, this exposure generates more hydrogen peroxide (H_2_O_2_), MDA, and ROS. Moreover, the activity of SOD is inhibited by PM2.5 exposure. This leads to abnormalities in autophagy and accelerates cellular aging, which makes it significant. It has been shown that Lycium barbarum polysaccharide (LBP) lessens the harmful effects of PM2.5 on cellular health. The senescence markers p16 and p21, which are raised as a result of PM2.5 exposure, are downregulated in HUVECs once autophagy defects are corrected, according to subsequent research. This finding underscores the capacity of LBP to attenuate cellular aging in HUVECs and highlights the significant regulatory role of autophagy in cellular senescence [90].

Vascular remodeling and the development of unstable atherosclerotic plaques are profoundly affected by vascular smooth muscle cell (VSMC) apoptosis [90], [91]. In pathological vascular conditions, oxidative stress is notably linked to the apoptosis of VSMCs. A recent hypothesis suggests that exposure to PM2.5 induces ROS formation, resulting in VSMC death. The thoracic aortas of rats exposed to PM2.5 showed reduced SOD activity and elevated MDA and ROS levels. Moreover, procyanidin intake significantly increased the expression of NRF2 and its associated antioxidant genes, such as heme oxygenase-1 (HO-1, also referred to as Hmox-1) and NADPH dehydrogenase (quinone) 1. Oxidative stress was ameliorated, and VSMC apoptosis was downregulated. Procyanidin successfully reduced the dose-dependent lethal effect of PM2.5 on VSMCs *in vitro* [92].

### Neuronal disorders

Numerous studies have highlighted the role of PM2.5 in the development of various neurological disorders, including ischemic stroke, neurodegenerative diseases, and epilepsy, through multiple RCD pathways [93], [94]. In particular, ferroptosis and autophagy have been extensively investigated, while other mechanisms, such as necroptosis, pyroptosis, and apoptosis, also contribute to neurological dysfunction associated with PM2.5 exposure.

#### PM2.5 and neurons

Cellular homeostasis is disrupted by oxidative stress, which is why exposure to PM2.5 is associated with certain neurological disorders. Neuronal cell lines Neuro-2a (N2a) and SH-SY5Y were utilized by Xiong et al. [95] to investigate the role of ferroptosis in neurotoxicity following exposure to PM2.5. The findings indicate that PM2.5 exposure induces ferroptosis in neuronal cells by disrupting iron metabolism, increasing oxidative stress, and impairing mitochondrial function. It downregulates the extracellular signal-regulated kinase (ERK)/cyclic AMP response element binding protein (CREB) pathway, reducing GPX4 expression, while ferroptosis inhibitors such as Fer-1 and deferoxamine restore GPX4 levels and improve cell survival. Among PM2.5 components, organic extracts show toxicity comparable to that of whole PM2.5, indicating they are the primary contributors to its neurotoxic effects [96]. We hypothesized that, under conditions of high doses and prolonged exposure to PM2.5, ferroptosis may represent the predominant mode of cell death. Additionally, the organic extract is likely to be the principal component within PM2.5 responsible for inducing ferroptosis.

Exposure to PM2.5 may increase ferroptosis in neuronal cells; however, the susceptibility of these cells to ferroptosis appears to be diminished. This presents challenges in interpreting the relationship between ferroptosis and neuronal cells exposed to PM2.5. In this regard, the functions of autophagy and the KEAP1-NRF2 signaling pathway in reducing PM2.5-induced cytotoxicity are the subject of recent research using mouse neuroblastoma N2a cells [11]. According to the study, exposure to PM2.5 disrupts lysosomal processes, such as lysosomal alkalinization, increased membrane permeability, and cathepsin B release, reducing autophagic flow. Moreover, dysregulated autophagy causes the p62-dependent activation of NRF2, which increases antioxidant gene expression and ferroptosis resistance. Because autophagic dysfunction inhibits their degradation, ferritin and GPX4, two essential ferroptosis-related proteins, accumulate and demonstrate reduced susceptibility to ferroptosis. The study revealed that the primary mechanism driving PM2.5-induced cytotoxicity in N2a cells is linked to cell death associated with lysosomal membrane permeabilization [11].

Exposure to PM2.5 has been found to contribute to microglial inflammatory responses by promoting ferroptosis and iron overload, mediated by the NRF2/HO-1 signaling pathway [97]. In mouse model studies, PM2.5 exposure has triggered microglial activation and subsequent neuroinflammation in the hippocampal regions. A microarray experiment revealed that genes associated with ferroptosis were significantly more prevalent in microglia exhibiting differentially expressed genes following exposure. Notably, exposure to PM2.5 for 24 h led to a substantial upregulation of HO-1. This upregulation, induced by NRF2, facilitated the breakdown of heme, consequently leading to ferroptosis and promoting iron accumulation. Importantly, the inhibition of NRF2 effectively prevented this pathological process [97].

It is well established that PM2.5 can stimulate ferroptosis; however, the mechanisms by which PM2.5 exposure leads to this process have been disputed by researchers [98]. One notable inconsistency relates to the expression of HO-1, which has been documented to exhibit both upregulation and downregulation following PM2.5 exposure [92], [97]. It is hypothesized that the effects of PM2.5 may be dose-dependent, where low doses promote the activation of the body’s antioxidative systems, particularly through the activation of NRF2 and HO-1 [97]. In contrast, exposure to higher doses is often associated with a reduction in these protective mechanisms, resulting in the downregulation of markers, such as HO-1 [92]. Another concept that may illuminate these contradictions is the consideration of susceptibility to ferroptosis in conjunction with its induction. In this context, the body responds to the dysfunction of autophagy following PM2.5 exposure by enhancing its antioxidative response, which includes the activation of NRF2 and GPX4 [11]. Nevertheless, it is important to note that GPX4 is frequently reported to be downregulated following such exposure [95].

#### The impact of PM2.5 on neurological disorders through pathways of cell death

**Fig. 5:**
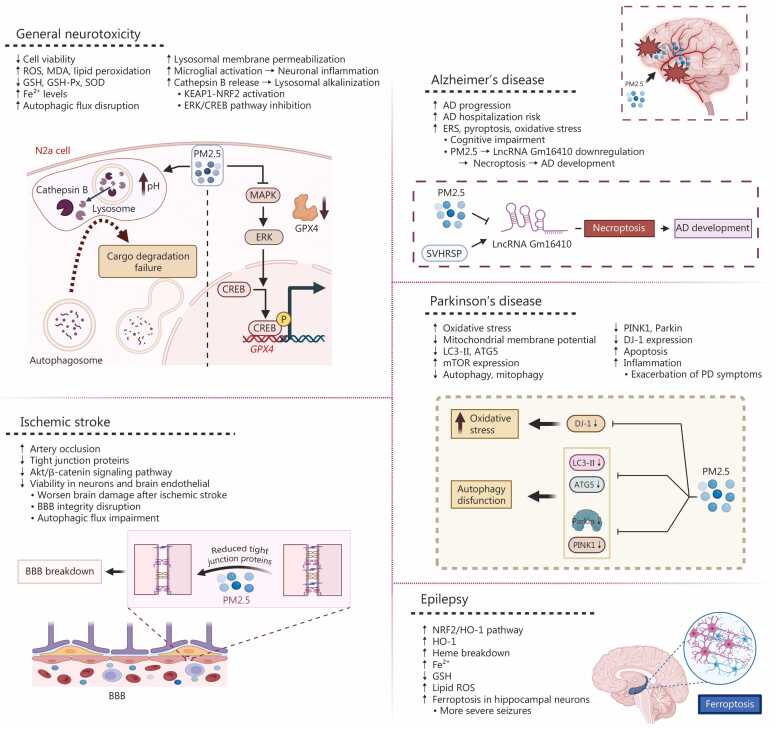
Schematic illustration of PM2.5-related neuronal disease and RCDs. Ischemic stroke is associated with artery occlusion, reduced tight junction proteins, BBB disruption, and autophagic flux impairment, leading to worsened ischemic brain damage. General neurotoxicity involves decreased cell viability, increased oxidative stress, elevated Fe²⁺ levels, and lysosomal membrane permeabilization, promoting microglial activation and neuronal inflammation through ERK/CREB pathway inhibition. In Alzheimer’s disease, PM2.5 exposure induces oxidative and endoplasmic reticulum stress, promoting pyroptosis and cognitive impairment via lncRNA Gm16410 downregulation and necroptosis. Epilepsy pathogenesis includes activation of the NRF2/HO-1 pathway, increased heme breakdown, iron accumulation, lipid ROS production, and ferroptosis in hippocampal neurons, aggravating seizure severity. In Parkinson’s disease, PM2.5 triggers oxidative stress, mitochondrial dysfunction, impaired autophagy and mitophagy, and reduced expression of DJ-1, LC3-II, ATG5, PINK1, and Parkin, thereby exacerbating dopaminergic neuronal loss and disease progression. Created with BioRender.com. ER. Endoplasmic reticulum; SVHRSP. Scorpion venom heat-resistant synthetic peptide; ROS. Reactive oxygen species; MDA. Malondialdehyde; GSH-Px. Glutathione peroxidase; SOD. Superoxide dismutase; KEAP1. Kelch-like ECH-associated protein 1; NRF2. Nuclear factor erythroid 2-related factor 2; ERK. Extracellular signal-regulated kinase; CREB. Cyclic AMP response element binding protein; MAPK. Mitogen-activated protein kinase; GPX4. Glutathione peroxidase 4; HO-1. Heme oxygenase-1; GSH. Glutathione; LC3-II. Microtubule-associated protein 1 A/1B-light chain 3-II; ATG. Autophagy-related; mTOR. Mechanistic target of rapamycin complex I; PINK1. PTEN-induced kinase 1; DJ-1. Deglycase protein DJ-1; RCD. Regulated cell death; BBB. Blood-brain barrier.

***Ischemic stroke*** Air pollution, especially high PM2.5 concentrations, greatly increases the risk of ischemic stroke. Recent research has investigated the mechanisms by which PM2.5 intensifies the consequences of ischemic stroke using both *in vitro* and *in vivo* models [95], [99]. Studies on Sprague-Dawley rats indicated that after middle cerebral artery occlusion, intravenous PM2.5 injection increased brain damage. The findings indicated a reduction in tight junction protein levels and alterations in autophagy flux within the brain tissue of these specimens. The study evaluated the effects of PM2.5 on ischemic brain injury in various cell types, including neurons, perivascular macrophages, and brain endothelial cells. HT-22 neurons, bEnd.3 endothelial cells and primary neurons exhibited notable decreases in viability when subjected to concurrent treatment with PM2.5 and oxygen-glucose deprivation [99]. It is particularly noteworthy that this co-treatment significantly inhibited the Akt/β-catenin signaling pathway and autophagy flux in HT-22 cells. Additionally, bEnd.3 cells subjected to oxygen-glucose deprivation in conjunction with PM2.5-preconditioned media revealed lower expression of tight junction proteins, while macrophages exposed to PM2.5 showed increased levels of metalloproteinase 9, which could exacerbate the blood-brain barrier (BBB) integrity [99] **(**Fig. 5**)**.

***Alzheimer’s disease*** Tau neurofibrillary tangles and beta-amyloid plaques are hallmark features of Alzheimer’s disease (AD), a progressive neurodegenerative disorder. Although some individuals may present with non-amnestic variants, AD is typically associated with profound amnestic cognitive impairments [95], [100]. Currently, dementia affects approximately 55 million people globally, and projections indicate this number could rise to over 100 million by 2050 [101]. Environmental factors, such as diet, lifestyle, and pollution, have been implicated as potential risk factors that may trigger epigenetic changes in genes related to AD [102]. Research has highlighted the impact of ambient air quality, notably through a longitudinal study revealing that each 5 µg/m^3^ increase in annual PM2.5 concentrations correlates with a 13% rise in the likelihood of hospitalization for AD [103]. This underscores the significant role that PM2.5 pollution may play in the exacerbation of AD progression. Chinese medicine has utilized scorpion venom for over a century, recognizing its potential to address viral infections, cancer, and epilepsy [104]. Current medical research emphasizes the bioactive peptides found in scorpion venom [scorpion venom heat-resistant peptide (SVHRP) and scorpion venom heat-resistant synthetic peptide (SVHRSP)] [102], [105], [106]. SVHRSP has shown promise in alleviating age-related cognitive decline by reducing oxidative stress and neuroinflammation, while SVHRP is known to protect dopaminergic neurons through the inhibition of critical cellular processes [107]. Although the precise role of these compounds in the advancement of AD induced by PM2.5 exposure remains uncertain, their anti-inflammatory properties suggest that they may represent viable therapeutic options for neurodegenerative disorders. Research conducted *in vivo* and *in vitro* has shown that exposure to PM2.5 leads to increased ERS, pyroptosis, and oxidative stress in models of AD. The findings suggest that PM2.5 exposure significantly worsens cognitive impairment, ERS, and pyroptosis in mice affected by AD. However, treatment with SVHRSP therapy has been found to mitigate the detrimental effects caused by PM2.5 exposure [108].

Necroptosis, a form of RCD, has recently gained considerable attention in the scientific community, particularly in relation to its connection with various biological processes mediated by long non-coding RNAs (lncRNAs) [109]. A recent study explores the role of lncRNA Gm16410 in neuronal necroptosis within the context of PM2.5-induced AD, as well as the potential therapeutic benefits of SVHRSP [110]. Experimental investigations utilizing AD animal models and cell cultures exposed to PM2.5 demonstrate that such exposure exacerbates the progression of AD by promoting necroptosis and downregulating lncRNA Gm16410. Behavioral assessments reveal that SVHRSP mitigates cognitive deficits in these subjects. Molecular assays indicate that lncRNA Gm16410 modulates necroptosis through the p38 mitogen-activated protein kinase (MAPK) signaling pathway in response to PM2.5 exposure. The findings suggest that SVHRSP may influence the development of AD by regulating lncRNA Gm16410 to attenuate necroptosis induced by PM2.5. From the perspective of lncRNA research, these results offer new insights into the mechanisms through which PM2.5 exposure accelerates the progression of AD [110] **(**Fig. 5**)**.

***Parkinson’s disease*** The second most common neurological condition after AD is Parkinson’s disease (PD), which affects 4% of people over 85 and 1%−2% of people over 65 [110], [111]. As the global population ages, it is anticipated that the prevalence of PD will rise, leading to significant economic challenges [112]. DJ-1 and PTEN-induced kinase 1 (PINK1) are two genetic variables that have been linked to the development of PD, while the exact etiology is yet unknown [110], [113]. Gene expression and the development of PD are thought to be significantly influenced by DNA methylation, and downregulating these genes may result in apoptosis and elevated oxidative stress [110], [114]. Emerging evidence indicates that exposure to PM2.5 disrupts the PINK1/Parkin signaling pathway, leading to mitochondrial dysfunction and exacerbation of PD-related neurodegeneration. Such findings suggest that individuals with PD may be particularly vulnerable to even short-term increases in PM2.5 levels [115]. Based on these observations, researchers have investigated how PM2.5 exposure affects the methylation and expression of PINK1 and DJ-1 in dopaminergic neurons of the substantia nigra. They employed rotenone to induce PD in murine models to explore the implications of PM2.5 exposure further. Following various exposure durations, they evaluated the methylation and expression of the relevant genes and found that prolonged exposure to PM2.5 markedly decreased *PINK1* and *DJ-1* mRNA levels while increasing the methylation of the cytosine-guanine (CpG) sites linked to these genes. The degradation of dopaminergic neurons was facilitated by PM2.5, which also made PD-related symptoms worse [115] **(**Fig. 5**)**.

Collectively, these findings highlight that PM2.5-induced epigenetic alterations in PINK1 and DJ-1 compromise mitochondrial homeostasis and dopaminergic neuron survival. Based on this molecular evidence, a subsequent study explored how PM2.5 exposure further aggravates neuronal damage and behavioral deficits through mitochondrial dysfunction and impaired autophagy. Exposure to PM2.5 has been shown to enhance apoptosis in PC12 cells treated with rotenone by elevating ROS levels and decreasing MMP. Furthermore, PM2.5 exposure reduced LC3-II and ATG5 expression levels while concurrently increasing mTOR expression, indicating suppression of autophagy. Notably, gene expression levels of mitophagy genes, specifically *PINK1* and *Parkin*, declined. In animal models of PD, inhalation of PM2.5 diminished behavioral impairments induced by rotenone, increased inflammatory mediators, and initiated oxidative stress and apoptosis within the substantia nigra. In PD models, rapamycin treatment was employed to mitigate the adverse effects of PM2.5 by promoting autophagy and mitophagy. The findings suggest that PM2.5 exposure exacerbates behavioral abnormalities linked to PD by increasing oxidative stress, decreasing autophagy and mitophagy, and inducing mitochondria-mediated neuronal death [116].

***Epilepsy*** People with epilepsy suffer from a common neurological disorder that has a substantial influence on their social interactions, behavior, learning, development, and self-esteem, among other aspects of their lives [117]. Recent research has investigated the potential correlation between exposure to PM2.5 and seizure activity and its contribution to ferroptosis in hippocampal neurons [17]. Findings indicated that disturbances in iron homeostasis and alterations in the NRF2-dependent ferroptosis pathway are more prominent in epileptic patients residing in areas with high PM2.5 concentrations than in those living in cleaner environments. In murine models, exposure to PM2.5 has been shown to exacerbate seizure symptoms and cognitive impairments, as evidenced by electroencephalogram measurements. The research explicitly used transmission electron microscopy imaging to demonstrate neuronal ferroptosis in the hippocampus and employed cell counting kit-8 (CCK-8) assays to reveal the neurotoxic effects associated with PM2.5 exposure. The relationship between PM2.5 exposure and hippocampal ferroptosis is further substantiated by increased Fe^2+^ and lipid ROS levels [17].

An important factor associated with PM2.5 that may exacerbate epilepsy is cadmium [118]. Research findings indicate that exposure to cadmium increases inflammatory responses and ferroptosis, potentially worsening seizure outcomes. The study has also identified significant human gene targets related to cadmium toxicity and its impact on seizure symptoms, emphasizing the relevance of the ferroptosis pathway and proposing melatonin as a prospective therapeutic agent. Additionally, the research illustrated that prolonged exposure to cadmium disrupts iron and lipid metabolism in the brain, subsequently triggering ferroptosis in the hippocampus. This disruption exacerbates seizure severity and anxiety-like behaviors in epileptic mice, highlighting the critical roles of neuroinflammation and ferroptosis in these conditions [118] **(**Fig. 5**)**.

### Lung diseases

**Fig. 6:**
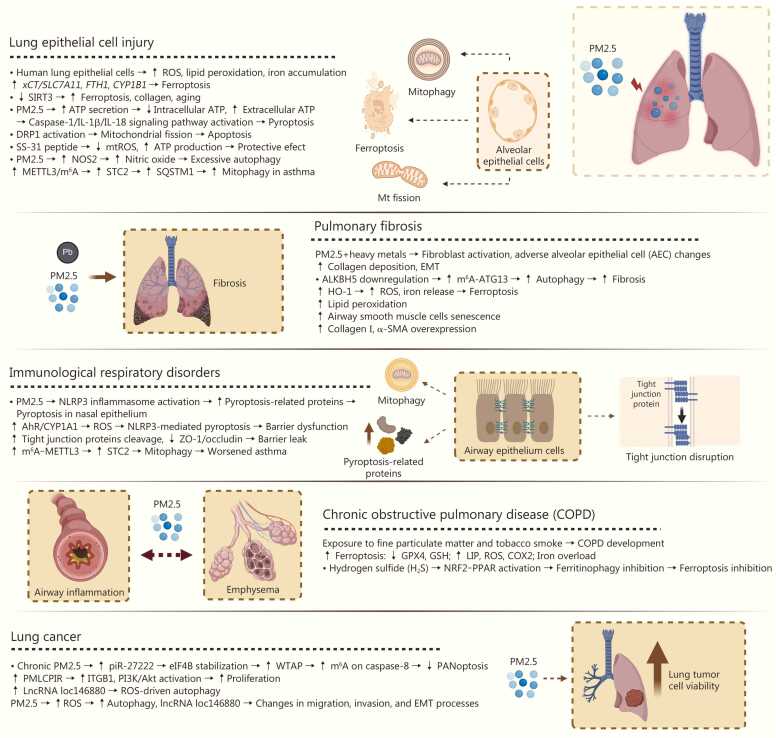
A schematic illustration of PM2.5-related pulmonary disorders and RCDs. Lung epithelial cells exposed to PM2.5 exhibit increased ROS, lipid peroxidation, iron accumulation, and ferroptosis mediated by genes like *xCT/SLC7A11*, *FTH1*, and *CYP1B1*, along with mitochondrial ROS elevation, membrane potential reduction, and ATP decrease. Mitochondrial fission via DRP1 activation leads to apoptosis, while excessive autophagy and mitophagy are induced through pathways involving NOS2, nitric oxide, and METTL3/m^6^A. Pulmonary fibrosis results from PM2.5 combined with heavy metals, stimulating fibroblast activation, EMT, autophagy, and collagen deposition, accompanied by ALKBH5 downregulation and ROS/iron-driven ferroptosis. Immunologically, PM2.5 activates the NLRP3 inflammasome, causing IL-1β and IL-18 release, pyroptosis, and barrier dysfunction in airway epithelium, linked to E-cadherin cleavage and tight junction disruption. Lung cancer progression involves piR-27222 and associated pathways that enhance proliferation, autophagy, and EMT. Finally, in COPD, PM2.5 exposure triggers ferroptosis via GPX4 depletion, oxidative stress, and iron overload, while hydrogen sulfide modulates NRF2-PPAR activation to inhibit ferroptosis and ferritinophagy. Created with BioRender.com. COPD. Chronic obstructive pulmonary disease; ROS. Reactive oxygen species; xCT. L-cystine/L-glutamate antiporter; SLC7A11. Solute carrier family 7 member 11; FTH1. Ferritin heavy chain 1; CYP1B1. Cytochrome p450 family 1 subfamily B member 1; SIRT3. Sirtuin 3; DRP1. Dynamin-related protein 1; SS-31. Mitochondria-targeted peptide antioxidant SS-31; mtROS. Mitochondrial reactive oxygen species; NOS2. Nitric oxide synthase 2; METTL3. Methyltransferase-like 3; m^6^A. N^6^-methyladenosine; STC2. Stanniocalcin 2; SQSTM1. Sequestosome 1; ALKBH5. AlkB homologue 5; ATG. Autophagy-related; HO-1. Heme oxygenase 1; α-SMA. α-smooth muscle actin; NLRP3. NLR family pyrin domain-containing 3; AhR. Aryl hydrocarbon receptor; CYP1A1. Cytochrome p450 family 1 subfamily A member 1; ZO-1. Zonula occludens-1; eIF4B. Eukaryotic initiation factor 4B; WTAP. Wilms tumor 1-associated protein; PMLCPIR. PM2.5-induced lung cancer upregulation of piRNA; ITGB1. Integrin subunit b 1; PI3K. Phosphoinositide 3-kinase; Akt. Protein kinase B; EMT. Epithelial-mesenchymal transition; GPX4. Glutathione peroxidase 4; LIP. Labile iron pool; COX2. Cyclooxygenase-2; NRF2. Nuclear factor erythroid 2-related factor 2; PPAR. Peroxisome proliferator activated receptor; RCD. Regulated cell death; IL. Interleukin.

Numerous pulmonary disorders are significantly linked to exposure to air pollutants, particularly PM2.5. These fine particles are known to contribute to the development of conditions such as pulmonary fibrosis, immunological respiratory disorders, COPD, and lung cancer **(**Fig. 6**)**. This association occurs through various RCD pathways, which will be examined in the following section.

#### Lung tissue cells

The human lung epithelial cells, a crucial component of the respiratory system, are adversely affected by air pollution. They serve as the initial barrier against different airborne contaminant particles [118], [119]. Research indicated that exposure to urban PM leads to a significant increase in the production of ROS and a corresponding decline in MMP. This exposure also induces supplementary ferroptotic symptoms, including lipid peroxidation, diminished antioxidant defenses, and intracellular iron accumulation. Oxidative stress is confirmed by increased levels of several markers, including CYP1B1 and FTH1. Moreover, significant elevations were seen in key ferroptosis-specific markers, such as the light chain subunit of the L-cystine/L-glutamate antiporter (*xCT*), *SLC7A11*, *FTL*, *FTH1*, and *CYP1B1*, suggesting that increased ROS levels induce oxidative stress and promote ferroptosis. Furthermore, there was a significant increase in lipid ROS and mtROS, which serve as indicators of lipid peroxidation [118], [120] **(**Fig. 6**)**. A recent study has examined the role of sirtuin 3 (SIRT3), an antioxidant mitochondrial deacetylase, specifically in relation to PM2.5-induced aging of pulmonary epithelial cells and ferroptosis [121]. The results showed that SIRT3 levels in AT2 cells and lung tissue were significantly decreased by PM2.5 exposure. Furthermore, *SIRT3* deficiency exacerbated inflammation and collagen accumulation within the lungs subjected to PM2.5 exposure. RNA sequencing analysis revealed a relationship between ferroptosis and cellular lifespan concerning the genes influenced by the depletion of SIRT3. Western blotting analyses demonstrated that AT2 cells deficient in SIRT3 exhibited increased levels of ferroptosis and proteins associated with cellular aging following PM2.5 treatment. Additionally, the overexpression of SIRT3 in PM2.5-treated AT2 cells reduced ferroptosis and indicators of aging [121].

Beyond the scope of ferroptosis, lung epithelial cells demonstrate a susceptibility to pyroptosis. Exposure to air pollution can induce pyroptosis in respiratory epithelial cells by activating the caspase-1/IL-1β/IL-18 signaling cascade and increasing LDH production. The increase in downstream inflammatory cytokines and chemokines, including IL-6, IL-8, chemokine (C-X-C motif) ligand 1 and 2, brought on by exposure to biofuel PM2.5 was successfully moderated by specific inhibitors that target caspase-1 (VX-765), as well as by the targeted siRNA reduction of IL-1β [122]. Notably, biofuel PM2.5 exposure resulted in an unprecedented increase in ATP secretion from intracellular compartments to extracellular spaces. This phenomenon may play a role in the inflammation and pyroptosis associated with PM2.5 by activating the caspase-1/IL-1β/IL-18 signaling pathway through autocrine and/or paracrine mechanisms. Furthermore, the induction of pyroptosis and the inflammatory response prompted by biofuel PM2.5 were significantly diminished by inhibiting ATP activity with apyrase or through the targeted siRNA knockdown of ATP receptors (*P2Y2* and *P2Y7*) [122].

Moreover, the function and presence of macrophages have a major impact on lung health [123]. Exposure to PM2.5 activates the NLRP3 inflammasome in macrophages, triggering pyroptosis that exacerbates oxidative stress, apoptosis, and inflammatory responses, thereby worsening lung injury [124]. Upon PM2.5 exposure, infiltration of leukocytes and macrophages increases in lung tissues, accompanied by elevated secretion of inflammatory cytokines, such as TNF-α and IL-6. Concurrently, levels of MDA, a marker of lipid peroxidation, rise significantly [125]. Meanwhile, the activity of antioxidant enzymes, including catalase, GPX, and SOD, decreases markedly, indicating oxidative imbalance. Further evidence of PM2.5-induced injury is provided by the analysis of apoptosis-related proteins, which reveal increased expression of the pro-apoptotic Bax and decreased expression of the anti-apoptotic Bcl-2, indicating enhanced apoptosis within lung tissues. Importantly, these pathological changes are significantly alleviated when NLRP3 is specifically deleted in macrophages. Mechanistically, PM2.5-induced pyroptosis is driven by activation of the NLRP3 inflammasome, as demonstrated by elevated levels of NLRP3, ASC, caspase-1, and GSDMD, along with increased secretion of IL-1β and IL-18, all of which are markedly reduced following NLRP3 deletion [124].

As previously stated, various cell death mechanisms are closely linked to mitochondria, making them an essential factor in the interpretation of cell deaths associated with PM2.5 exposure. The pathophysiology of lung disorders is profoundly influenced by mitochondrial function, which exhibits increased susceptibility to exposure to PM2.5 [126]. The study conducted by Liu *et al*. [125] examined the effects of varying concentrations of PM2.5 on human lung epithelial cells. The findings revealed the presence of PM2.5 components within the cytoplasm, which substantially damaged mitochondrial structures. Additionally, a decrease in ATP production and cellular respiration occurred along with calcium excess. Furthermore, the translocation and phosphorylation of dynamin-related protein 1 (DRP1) revealed that exposure to PM2.5 altered the kinetics of mitochondrial fission and thereby triggered an apoptotic mechanism driven by mitochondria. Mitochondrial division inhibitor 1 (Mdivi-1), a DRP1 inhibitor, reversed the activated mitochondrial apoptotic pathway, therefore substantially minimizing the pro-apoptotic effects caused by PM2.5. In addition, exposure to PM2.5 reduced the mitochondria’s oxygen consumption and glycolysis rates. D-Arg-Dmt-Lys-Phe-NH2 (SS-31, also referred to as elamipretide and MTP-131) is a remarkable peptide that has the power to penetrate cells, specifically reaching the inner mitochondrial membrane. It works to reduce the accumulation of mtROS in a dose-dependent manner, effectively neutralizing ROS and oxygen-free radicals [126], [127]. It has been shown that the mitochondria-targeted peptide SS-31 effectively eliminates mtROS while raising ATP [128].

Ultimately, the exploration of autophagy linked to PM2.5-exposed lung tissue opens new avenues for understanding resilience and adaptation in the face of environmental challenges. PM2.5 exposure specifically triggered the expression of nitric oxide synthase 2 (NOS2) and the resulting nitric oxide generation, which increased excessive autophagy. Eventually, the disruption of NOS2 signaling pathways successfully reduced the production of autophagosomes and the cellular death they caused [129].

#### Pulmonary fibrosis

PM2.5 and exposure to atmospheric heavy metals have been linked to pulmonary fibrosis [130]. According to research, exposure to PM2.5 causes fibrotic alterations in alveolar epithelial cells (AECs) via activating paracrine signaling pathways in lung fibroblasts [131]. Among the heavy metals that were studied, lead (Pb), cadmium, manganese (Mn), and arsenic have all shown unique impacts on the behavior of lung fibroblasts. The migration, proliferation, and survival of lung fibroblasts are adversely affected by prolonged exposure to these metals. Through the activation of the ERK signaling pathway, Mn has been shown to raise the levels of fibrotic markers in lung fibroblasts. However, conditional media derived from fibroblasts treated with Mn did not induce fibrotic changes in AECs. Conversely, conditioned media from Pb-exposed fibroblasts significantly increased the levels of fibrosis and apoptotic markers in AECs through the stimulation of the ERK pathway [131]. This indicates that heavy metals can induce various cellular responses depending on whether their effects are direct or mediated through secreted factors that impact AECs.

Autophagy is a well-documented form of RCD that contributes to the development of fibrosis [132]. In order to elucidate its underlying mechanisms, research has demonstrated that the deletion of AlkB homologue 5 (*ALKBH5*) in murine models significantly accelerates inflammation, autophagy, and the progression of pulmonary fibrosis. Notably, in lung epithelial cells, there is a marked increase in the N^6^-methyladenosine (m^6^A) modification of *ATG13* mRNA at position 767, attributable to the downregulation of the ALKBH5 protein. This specific m^6^A modification is critical in promoting autophagy, which subsequently aggravates inflammatory responses, particularly after exposure to fine PM, such as PM2.5 particles. These findings highlight the intricate interplay between ALKBH5, autophagy processes, and inflammatory pathways in the context of pulmonary health [133].

The overexpression of HO-1 has been identified through proteomic analysis as a significant contributor to lung fibrosis and ferroptosis associated with PM2.5 exposure [134]. The facilitation of heme-containing protein degradation by HO-1 results in reduced mitochondrial activity and increased production of ROS within fibrotic cells, accompanied by iron release. Transmission electron microscopy has shown that PM2.5 causes major alterations in mitochondrial morphology, including reductions in size and increases in membrane density, by penetrating the mitochondria of these cells. It has been shown that the HO-1 inhibitor zinc protoporphyrin and 2-(2,2,6,6-tetramethylpiperidin-1-oxyl-4-ylamino)-2-oxoethyl (mito-TEMPO), a mitochondrion-targeted antioxidant, lessen the ferroptosis and fibrosis induced by PM2.5 [18]. In this study, ferroptosis was associated with the AMPK-unc-51-like autophagy activating kinase 1 (ULK1) pathway-mediated activation of autophagy and NCOA4-mediated ferritin breakdown in fibrotic cells. Autophagy suppression with 3-methyladenine or an AMPK inhibitor, alongside *NCOA4* knockdown, resulted in decreased lipid peroxidation and iron buildup. Notably, PM2.5 induced the epithelial-mesenchymal transition (EMT) and apoptosis of fibrotic cells [18].

Airway smooth muscle cells (ASMCs) play a critical role in airway remodeling, which has a substantial impact on the pathogenesis of asthma and COPD [135]. The effect of PM2.5 on ASMCs has been studied recently. Researchers found that the quantity of collagen and smooth muscle deposited *in vivo* increases when inhaled PM2.5 particles accumulate in the airway smooth muscle bundles. According to *in vitro* research, PM2.5 causes both rat and human ASMCs to overexpress collagen I and α-smooth muscle actin (α-SMA). Furthermore, a senescence-associated secretory phenotype is triggered by exposure to PM2.5, which causes cellular senescence in these ASMCs via an autophagy-mediated signaling cascade that involves GATA binding protein 4 (GATA4), TNF receptor-associated factor 6 (TRAF6), and nuclear factor κB (NF-κB). Ultimately, this signaling cascade enhances the synthesis of collagen I and α-SMA, contributing to smooth muscle remodeling within the airways [136]_._

#### Immunological respiratory conditions

The induction of inflammation by PM2.5 significantly exacerbates respiratory conditions, such as allergic rhinitis (AR) [136], [137]. Yuan *et al*. [138] investigated the role of PM2.5 in AR through the induction of pyroptosis in nasal epithelial cells. Clinical research has demonstrated that patients with AR exhibit NLRP3 inflammasome activation and elevated pyroptosis-related proteins in their nasal mucosa compared to control subjects. Both *in vitro* and *in vivo* studies indicated that exposure to PM2.5 enhances these markers, which damage tight junction proteins and compromise the integrity of the inner barrier. Additionally, PM2.5 increases the AhR pathway, which in turn increases CYP1A1 transcription and thereby ROS generation. Remarkably, the protective benefits linked to AhR downregulation decreased PM2.5-induced pyroptosis and barrier dysfunction. This highlights how the AhR/CYP1A1/ROS/NLRP3 pathway plays a critical role in mediating the damage to the epithelium caused by PM2.5 exposure [138].

Asthma is a chronic inflammatory condition that affects millions of individuals globally. It is influenced by both genetic and environmental factors, including exposure to allergens [138], [139]. Airway hyperresponsiveness (AHR) is a key feature of asthma characterized by an increased sensitivity of the airways to inhaled constrictor agonists. This condition is marked by a heightened maximum response to these agonists and a steeper slope in the dose-response curve [140]. A recent study indicates that exposure to PM2.5 may exacerbate AHR, with these effects linked to inflammation resulting from necroptosis. Interestingly, anti-Fas treatment has demonstrated the potential to reduce the inflammation and necroptosis associated with PM2.5 exposure, offering a promising avenue for managing AHR [141].

The airway epithelial barrier, which serves to protect against infections and allergens, depends on the integrity of adhesion junctions and tight junctions [140], [142]. Adherens junctions, such as E-cadherin, are essential for facilitating cell adhesion. Tight junctions, composed of proteins including occludin, claudins, and zonula occludens-1 (ZO-1), establish a selective barrier that regulates permeability [140], [143]. An increase in apoptosis may undermine this barrier’s function. Apoptosis can disrupt tight junctions through caspase cleavage, impacting various health issues [144]. Research indicates that PM2.5 exacerbates impairment in asthmatic murine models by promoting epithelial cell apoptosis through the Fas-associated via death domain (FADD)-mediated pathways and decreasing the expression of crucial tight junction proteins, including ZO-1, occludin, claudins, β-catenin, and E-cadherin, thereby compromising barrier integrity and function [145]. A reduction in FADD expression leads to decreased apoptosis and a restoration of tight junction protein levels, suggesting that PM2.5 may elevate epithelial cell mortality through a FADD-dependent mechanism [145].

Mitophagy is essential for maintaining cellular health and is linked to inflammatory conditions, such as asthma. Inhibition of mitophagy may mitigate allergic airway inflammation; however, its dysregulation exacerbates inflammation and compromises the integrity of the epithelial barrier [146]. Furthermore, stanniocalcin 2 (STC2), a secreted glycoprotein hormone, plays a critical role in regulating oxidative stress, inflammation, and angiogenesis [146], [147]. In this regard, a recent study identified that PM2.5 exposure elevates m^6^A methylation of *STC2* mRNA via the methyltransferase methyltransferase-like 3 (METTL3), which binds to YTH N^6^-methyladenosine RNA-binding protein F2 (YTHDF2) to promote m^6^A-dependent mRNA stability, thereby enhancing STC2 expression and activating mitophagy. Elevated STC2 subsequently exacerbates asthma severity and enhances mitochondrial autophagy through the upregulation of sequestosome 1 (SQSTM1). These findings indicate that serum levels of METTL3 and STC2 exhibit a correlation with PM2.5 exposure. This suggests that these biomarkers could potentially serve as indicators for asthma exacerbations triggered by PM2.5 pollutants [148].

#### Chronic obstructive pulmonary disease

COPD is a progressive respiratory condition marked by enduring symptoms and restricted airflow [148], [149]. Pulmonary emphysema, a severe variant of COPD, compromises the airways and alveoli, leading to the degradation of alveolar walls and impaired gas exchange. Dyspnea, chronic cough, and reduced exercise capacity are symptoms indicative of this decrease, significantly adversely affecting individuals’ quality of life [148], [150]. Exposure to fine PM and tobacco smoke is a significant risk factor for the development of COPD [151]. In COPD patients and animal models exposed to PM, anti-oxidative markers, such as GPX4 and GSH, were diminished, whereas oxidative factors like labile iron pool (LIP), ROS, cyclooxygenase-2 (COX2), and MDA were elevated, with an accumulation of active iron [152]. Notably, pretreatment with sodium hydrosulfide (exogenous hydrogen sulfide) effectively reduced airway inflammation and emphysema by inhibiting ferroptosis. This study suggests that hydrogen sulfide may mitigate PM-induced emphysema and airway inflammation by restoring redox equilibrium and inhibiting ferroptosis via activating the NRF2-peroxisome proliferator-activated receptor (PPAR), thereby inhibiting the ferritinophagy signaling pathway [152] **(**Fig. 6**)**.

#### Lung cancer

Small non-coding RNAs, including microRNAs and P-element-induced wimpy testis-interacting RNAs (piRNAs), assume a crucial role in various disorders [148], [153], [154]. piRNAs, which range in length from 26 to 32 nucleotides, inhibit transposable elements and maintain genomic integrity in germ cells through mechanisms of DNA methylation [155]. Studies have documented the presence of piRNAs in somatic cells, suggesting their potential involvement in human diseases [156], [157], [158]. Due to their stability, piRNAs may serve as promising biomarkers for disease incidence [159]. The most prevalent RNA modification associated with disease is m^6^A, which influences mRNA stability, splicing, and translation [160]. Recent research on the epigenetic mechanisms underlying PM2.5-induced lung cancer has identified piR-27222 as an oncogene that modulates cell death via alterations in m^6^A. This oncogene is upregulated by long-term exposure to PM2.5. piR-27222 stabilizes and deubiquitinates the eukaryotic initiation factor 4B (eIF4B) through direct binding, simultaneously reducing its interaction with Parkinson protein 2. Increased expression of eIF4B and thereby elevated levels of Wilms tumor 1-associated protein (WTAP) enhance the m^6^A modification of the caspase-8 transcript. Consequently, the stability of *caspase-8* mRNA is diminished, contributing to the resistance of lung cancer cells to PANoptosis [25], [159]. PM2.5-induced lung cancer upregulation of piRNA (PMLCPIR) represents another identified piRNA that promotes the proliferation of lung cancer cells and PM2.5-transformed cells [161]. RNA sequencing has revealed that PMLCPIR targets the integrin subunit b 1 (ITGB1), leading to an increased expression of this protein and the activation of the PI3K/Akt signaling pathway through its interaction with nucleolin. The inhibition of PMLCPIR may induce apoptosis in lung cancer cells, thereby increasing their susceptibility to anti-tumor therapies [161].

LncRNAs also play a significant role in the development of lung cancer through pathways associated with cell death and the contributions of short non-coding RNAs. When lung cancer cells are exposed to PM2.5, there is an increase in ROS levels, which triggers autophagy mechanisms. The elevated ROS leads to an upregulation of the lncRNA designated loc146880. This specific lncRNA is associated with promoting autophagy in A549 cells. Key cellular processes, such as migration, invasion, and EMT, are significantly influenced by the interplay between heightened ROS levels and loc146880 [162] **(**Fig. 6**)**.

### Male infertility

Male infertility represents a significant global challenge, impacting approximately 8% of men, with around 50% of these cases identified as idiopathic [163]. Chronic inhalation of PM2.5 has been linked to disorders of spermatogenesis and infertility; however, the precise molecular mechanisms underlying these effects remain inadequately understood [164]. Furthermore, testosterone, an essential reproductive hormone produced by Leydig cells, is susceptible to disruption by various environmental pollutants, thereby exacerbating the male infertility issue [163], [165]. This section will examine the implementation of different cell death mechanisms in fertility-related cells affected by PM2.5.

#### Leydig cells and spermatocytes

In male animals, testosterone is synthesized and secreted by Leydig cells. These cells are critical to male reproductive health and can be influenced by environmental contaminants, which have the potential to alter testosterone levels. It is imperative to maintain stable testosterone levels for overall reproductive health [163]. A notable aspect of aberrant autophagy associated with PM2.5-induced reductions in testosterone synthesis is governed by the METTL3-m^6^A-silent information regulator Sirtuin 1(SIRT1) axis. Long-term exposure to PM2.5 has been shown to alter testicular morphology, increase sperm abnormalities, and decrease sperm count in an *in vivo* study. These alterations diminished testosterone levels within the testes and subsequently in the bloodstream. *In vitro* exposure to PM2.5 increased m^6^A modifications and elevated METTL3 levels, leading to impaired testosterone production and failures in autophagy within Leydig cells. Inhibition of METTL3 resulted in enhanced testosterone synthesis and improved autophagic processes. Furthermore, PM2.5 mechanistically contributed to aberrant autophagy by intensifying the m^6^A modification of *SIRT1* mRNA in Leydig cells. Administration of the SIRT1 activator SRT1720 augmented autophagy and facilitated a further increase in testosterone production [166]. A recent study investigated the effects of PM2.5 on testicular function utilizing primary Leydig cells and conditional knockout mice. Following PM2.5 exposure, *Sirt1*-mutant and wild-type mice showed reduced serum testosterone levels, vacuolization of Leydig cells, structural damage to seminiferous tubules, and a decline in sperm quality. The hypoxia-inducible factor 1 (HIF-1) signaling pathway, ferroptosis, and the production of steroid hormones in the testes after exposure to PM2.5 were all associated with differentially expressed genes, according to enrichment studies. Similar responses were observed in primary Leydig cells treated with PM2.5. Furthermore, *Sirt1* knockdown in response to PM2.5 exposure resulted in enhanced expression and activation of HIF-1α, which correlated with changes in cellular iron levels, oxidative stress markers, and ferroptosis indicators. In conclusion, exposure to PM2.5 activates the SIRT1/HIF-1a signaling pathway, leading to ferroptosis that impedes male testosterone production [20].

**Fig. 7:**
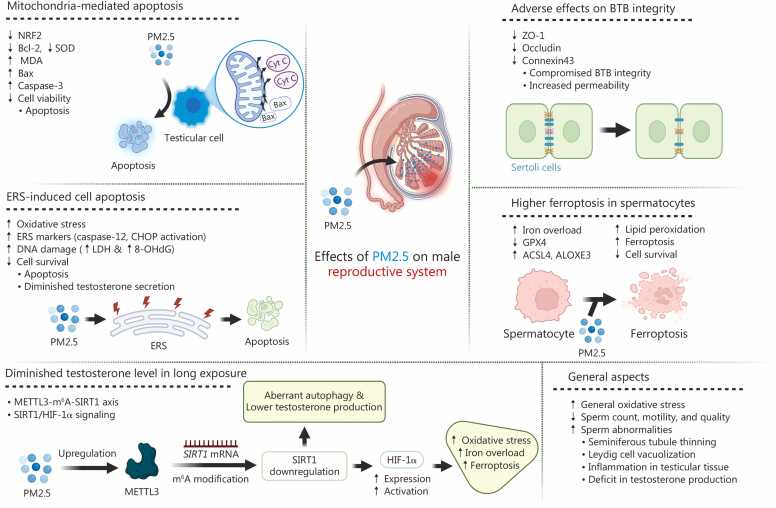
Schematic illustration of PM2.5-related infertility and RCDs. PM2.5 induces mitochondria-mediated apoptosis in testicular cells, characterized by reduced NRF2 and antioxidant enzymes, increased Bax, caspase-3 activation, and cytochrome C (Cyt C) release, resulting in cell death. Endoplasmic reticulum stress (ERS) triggered by PM2.5 leads to oxidative stress, DNA damage, apoptosis, and reduced testosterone secretion. PM2.5 impairs BTB integrity by downregulating occludin, ZO-1, and connexin 43 in Sertoli cells, increasing permeability. In spermatocytes, PM2.5 promotes ferroptosis through iron overload, lipid peroxidation, and modulation of ferroptosis regulators GPX4, ACSL4, and ALOXE3, decreasing cell survival. Long-term exposure diminishes testosterone via the METTL3-m^6^A-SIRT1 axis, where PM2.5 upregulates METTL3, leading to m^6^A-mediated *SIRT1* mRNA modification and downregulation, which activates HIF-1α expression and promotes oxidative stress, iron overload, and ferroptosis. General pathological aspects include oxidative stress, reduced sperm count, motility, and quality, seminiferous tubule thinning, Leydig cell vacuolization, inflammation in testicular tissue, and impaired testosterone production. The figure comprehensively depicts how PM2.5 damages male reproductive health through apoptosis, ferroptosis, barrier disruption, and hormonal dysregulation. Created with BioRender.com. BTB. Blood-testis barrier; RCD. Regulated cell death; NRF2. Nuclear factor erythroid 2-related factor 2; MDA. Malondialdehyde; BCL-2. B-cell lymphoma-2; Bax. Bcl-2 associated X protein; ER. Endoplasmic reticulum; CHOP. C/EBP homologous transcription factor protein; 8-OHdG. 8-hydroxydeoxyguanosine; LDH. Lactate dehydrogenase; ZO-1. Zonula occludens-1; GPX4. Glutathione peroxidase 4; ACSL4. Acyl-CoA synthetase long-chain family member 4; ALOXE3. Arachidonate lipoxygenase 3; METTL3. Methyltransferase-like 3; m^6^A. N^6^-methyladenosine; SIRT1. Silent information regulator Sirtuin 1; HIF-1α. Hypoxia-inducible factor 1α; SOD. Superoxide dismutase.

In another study, male Wistar rats were subjected to exposure to PM2.5 for 12 weeks, utilizing a concentrated animal whole-body exposure system. Reduced sperm quality, oxidative stress, ERS, inflammation, apoptosis, and testicular tissue damage were all brought on by PM2.5 exposure. PM2.5 treatment decreased cell survival, increased ROS, and increased LDH and 8-hydroxydeoxyguanosine (8-OHdG), leading to DNA damage, ERS, and apoptosis, according to *in vitro* studies conducted on primary testicular spermatogonia and Leydig cells. Additionally, exposure to PM2.5 prevented Leydig cells from secreting and synthesizing testosterone. The findings demonstrated that oxidative stress activates ERS pathways, which subsequently stimulate the activation of caspase-12 and C/EBP homologous transcription factor protein (CHOP), both associated with apoptosis. However, applying ERS inhibitors and antioxidants significantly mitigated these detrimental effects [167]. Spermatocytes, which are also significant contributors to fertility, may be severely affected by PM2.5 exposure [168]. Significant pathogenic damage and abnormal mitochondria were observed in spermatocytes within an animal model subjected to real-time exposure to PM2.5. Key indicators of ferroptosis and iron metabolism exhibited notable alterations in testicular tissues, alongside a reduction in cell viability observed *in vitro*. Transcriptome analysis indicated a substantial enrichment of the ferroptosis pathway. Lipid peroxidation and iron overload were verified to be present in spermatocytes exposed to PM2.5. *Gpx4*, *Acsl4*, and *Aloxe3* are significant target genes. The use of the iron chelator deferoxamine and the lipid peroxidation inhibitor Fer-1 may, crucially, lessen the negative effects of PM2.5 exposure, highlighting the role ferroptosis plays in PM2.5 spermatocyte toxicity [168] **(**Fig. 7**)**.

#### Blood-testis barrier

An integral part of the male reproductive system, the blood-testis barrier (BTB) creates the ideal conditions for spermatogenesis via the combined action of tight junctions, adherens junctions, and gap junctions. This intricate wall regulates the testicular environment and protects sperm cells from contaminants [169]. A recent study has shown that the integrity of the BTB is adversely affected by exposure to PM2.5 and other small PM. Following such exposure, the expression of important tight junction proteins, including ZO-1 and occludin, is significantly decreased. Furthermore, connexin 43, a crucial protein for gap junction interaction, has shown a sharp decline in levels. The general state of reproductive health and male fertility may be greatly impacted by these disturbances [170].

Exposure to PM2.5 has also been shown to impair Sertoli cell function, which is essential for spermatogenesis and the integrity of the BTB. The consequent dysfunction of Sertoli cells disrupts the testicular microenvironment and compromises germ cell development [171]. Recent evidence indicates that the enzyme NAD(P)H quinone oxidoreductase 1 (NQO1) exerts a protective effect against PM2.5-induced cytotoxicity by modulating the mitochondrial unfolded protein response (UPRmt) pathway. Specifically, NQO1 overexpression enhances cellular antioxidant defense mechanisms, as evidenced by elevated SOD activity, decreased MDA accumulation, and upregulation of UPRmt-associated proteins, including heat shock protein 60 (HSP60), activating transcription factor 5 (ATF5), and mitochondrial caseinolytic protease P. In contrast, NQO1 deficiency disrupts mitochondrial function, reflected by decreased MMP and increased cytosolic cytochrome C release due to suppression of UPRmt signaling. This mitochondrial dysfunction subsequently elevates the Bax/Bcl-2 ratio, thereby promoting apoptotic signaling pathways in testicular cells. Collectively, these findings demonstrate that the NQO1-mediated activation of the UPRmt axis maintains mitochondrial protein balance and redox homeostasis, effectively mitigating PM2.5-induced oxidative stress and apoptosis in germ cells [171] **(**Fig. 7**)***.*

#### Developmental outcomes

Researchers looked at how male offspring’s reproductive health was affected by the mother’s exposure to PM2.5 [172]. To examine it, mice were exposed intratracheally to PM2.5 every 3 d throughout pregnancy. Mothers exposed to PM2.5 during pregnancy were found to have testicular damage in their offspring. This damage manifested as decreased levels of androgen-binding protein and testosterone, a reduction in primary spermatocytes and spermatids, compromised sperm quality and quantity, and a decrease in seminiferous tubule diameter. Further findings included an upregulation of apoptosis and activation of the inositol-requiring transmembrane kinase/endoribonuclease 1 (IRE-1)/phosphorylated c-Jun N-terminal kinase (p-JNK)/cleaved caspase-12/cleaved caspase-3 signaling pathway [172]. Bcl-2, NRF2, and SOD were all found to decrease in response to PM2.5 exposure. HSP60 is downregulated by such exposure, while ATF5 is stimulated. Concurrently, there is an observed increase in MDA, Bax, and caspase-3 levels, which could better explain the process of apoptosis induction through PM2.5 exposure [172], [173]. In this study, it is necessary to address a complex and controversial question regarding the differential effects of HSP60 and ATF5, both of which are components of the UPRmt, in the context of exposure to PM2.5. Although this question remains unanswered by the authors, we propose a hypothesis to elucidate this phenomenon. The heightened oxidative stress resulting from PM2.5 exposure may lead to significant mitochondrial damage, which could reduce HSP60 levels, a mitochondrial protein, due to the depletion of mitochondrial chaperone capacity. Subsequently, a compensatory signaling pathway may be activated through the upregulation of ATF5, aimed at facilitating mitochondrial repair mechanisms. Presently, there is a lack of studies about the impact of PM2.5 on the reproductive systems of young children. Conversely, the reproductive systems of young children are more susceptible to environmental factors, potentially resulting in issues with spermatogenesis in adulthood [174]. PM2.5 induces reproductive toxicity and spermatogenesis dysfunction in male rats via promoting oxidative stress, mitochondrial dysfunction, mitochondrial damage, and a rise in cell death forms like apoptosis in spermatogenic cells [173], [175], [176]. According to an experiment conducted by Wang *et al*. [173], PM2.5 disrupts the UPRmt, which leads to spermatogenesis failure during sexual maturity in young male Sprague-Dawley rats. The findings indicated that greater PM2.5 doses significantly reduced the quantity of spermatogenic cells and the rates of conception. These cells showed vacuolated, enlarged, and degraded mitochondria, and the PM2.5-exposed groups featured higher rates of apoptosis than the control groups. Disruption of UPRmt-related proteins (CHOP, HSP60, ATF4, and ATF5) was observed upon exposure to PM2.5. In prepubertal rats, PM2.5 led to male infertility by causing oxidative stress, disrupting UPRmt, impairing mitochondria, and increasing apoptosis in spermatogenic cells. As mentioned, the enzyme NQO1 is crucial for reducing PM2.5 toxicity by regulating the UPRmt pathway [173]. Therefore, it is expected that utilizing an NQO1 agonist may be a promising strategy to combat infertility in adolescents exposed to PM2.5 **(**Fig. 7**)**.

### Liver

Liver fibrosis is a common pathological disease that may result from two serious liver disorders, cirrhosis and liver cancer. This process, which is known as a reversible injury-repair mechanism, is mostly initiated by hepatic stellate cells (HSCs). Upon activation, HSCs differentiate into fibroblasts or myofibroblasts that express large quantities of α-SMA, undergoing significant morphological and functional changes. Due to the enhanced collagen formation, proliferation, and differentiation of these activated cells, this reaction causes an imbalance between the deposition and breakdown of extracellular matrix (ECM) [177]. Excess production and accumulation of ECM proteins, mainly collagen and fibronectin, are what cause liver fibrosis [178], [179]. The development and progression of liver fibrosis are significantly influenced by oxidative stress and ROS [180]. Mitochondria, which generate energy, simultaneously produce ROS that can accumulate and result in cellular damage [181]. Regulation of ROS-mediated mitochondrial damage may, in turn, lead to liver fibrosis, particularly in response to PM2.5 [182]. Mechanistically, PM2.5 was shown to activate LX-2 cells and primary HSCs by downregulating gelatinases like matrix metalloproteinase 2 and upregulating myofibroblast markers like collagen type I and α-SMA, thereby causing liver fibrosis. Moreover, PM2.5 modified enzymatic antioxidant levels and stimulated mitochondrial fission. There was evidence that the severity of liver fibrosis may be lessened by preventing the increased ROS that PM2.5 produces. Furthermore, PM2.5 activated the PINK1/Parkin pathway to trigger mitophagy [182]. According to *in vitro* research, melatonin increases the expression of NRF2 and its downstream target genes, which reduces the generation of ROS and mitochondrial damage brought on by PM2.5 exposure. Additionally, melatonin effectively reduces oxidative stress and the elevation of fibrogenic markers associated with PM2.5 exposure. Notably, LX-2 cells treated with siNRF2 did not demonstrate the antifibrotic effects typically observed with melatonin. *In vivo* experiments involving mouse hepatocytes subjected to PM2.5, mitochondrial defects, and fragmentation were observed, accompanied by increased levels of Parkin and PINK1. These results imply that melatonin protects against oxidative stress caused by PM2.5 by partly reducing these negative effects and activating the NRF2 signaling pathway. Moreover, it contributes to the decrease in inflammation and liver lesions. The protective benefits of melatonin were significantly diminished in *NRF2*-knockout animals exposed to PM2.5, and they showed more severe inflammation and liver fibrosis than wild-type mice [183].

Persistent oxidative stress brought on by long-term ethanol intake damages liver tissue and eventually results in fibrosis [184]. According to recent research, oxidative stress is exacerbated by ambient PM2.5 exposure, which may exacerbate liver fibrosis linked to a high-fat diet [185]. Nevertheless, the precise processes driving these processes remain largely unknown. Researchers investigated the correlation between ethanol consumption and exposure to ambient PM2.5 in mice over a 12-week duration. The findings revealed significant increases in mtROS levels and hepatic oxidative stress due to ethanol intake, alongside the upregulation of profibrotic markers, such as transforming growth factor-β1 (TGF-β1) and collagen I. Furthermore, the study reported the induction of hepatic ferroptosis and an enhancement of liver fibrosis. Notably, these adverse effects were intensified by the simultaneous exposure to ethanol and ambient PM. *In vitro* tests using LX-2 cells exposed to PM2.5, either alone or in combination with ethanol, showed significant upregulation of TGF-β1 and collagen I, increased levels of mtROS, ferroptosis-related proteins, oxidative stress markers like 4-hydroxynonenal, and a change in the GSH/oxidized GSH (GSSG) ratio [185].

Exposure to PM2.5 is also linked to both the incidence and death rates of hepatocellular carcinoma (HCC) [186]; however, little is known regarding the processes by which PM2.5 affects HCC cell growth. Research has examined the impact of PM2.5 on the stem cell-like properties of HCC cells. Findings indicate that PM2.5 significantly enhances the stemness of these cells. Furthermore, *in vivo* studies revealed that exposure to PM2.5 increased proliferation, metastasis, and EMT of HCC cells. Additional investigations demonstrated that PM2.5 induces the production of ROS, which subsequently elevates stemness. This mechanistic pathway involves the upregulation and translocation of NRF2, driven by a reduction in KEAP1 due to heightened ROS levels. This sequence of events triggers autophagy, thereby further augmenting the stemness of HCC cells [187]. We have discussed several studies that indicate a protective effect of NRF2; this study provides evidence that NRF2 may induce cancer cell stemness by stimulating autophagy. This potential oncogenic property should be carefully considered when developing pharmacological interventions, especially given that NRF2 is well-documented for its antioxidant properties.

In the field of oncology, the significance of detection is paramount, complementing treatment efforts and enhancing our understanding of the disease. Despite the challenges presented by air pollutants and the lack of reliable hub markers, there exists a compelling opportunity for innovation. By adopting advanced methodologies, such as liquid biopsies and microfluidics, we can identify critical biomarkers associated with PM2.5 and elucidate the mechanisms by which these pollutants may contribute to cancer development. Through collaborative efforts, we can transform challenges into significant advancements in the understanding and management of cancer [188].

### Renal disease

In a research study investigating the effects of PM2.5 on renal health, researchers subjected C57BL/6 mice to PM2.5 exposure for 12 weeks. Subsequently, urine, blood, and kidney tissues were collected to evaluate kidney function and identify any pathological alterations associated with PM2.5 exposure. The findings revealed substantial damage to the glomeruli and renal tubules in the kidneys of the exposed mice. Further analysis conducted through mRNA sequencing demonstrated a role for pyroptosis in this process. The researchers observed elevated expression levels of key inflammatory markers, including IL-1β, GSDMD, caspase-1, and NLRP3. There was also evidence of pyroptosis and activation of the NLRP3 inflammasome in both the kidney tissues and Bumpt cells exposed to PM2.5. Importantly, the study indicated that the application of NLRP3 and caspase-1 inhibitors significantly improved the condition of Bumpt cells affected by PM2.5, thereby suggesting potential therapeutic strategies for mitigating the adverse effects of PM on renal health [21].

It has also been shown that exposure to PM2.5 causes considerable mitochondrial damage in renal tubular cells, which manifests as reduced ATP generation, increased oxidative stress, decreased membrane potential, and mitochondrial calcium excess. One important element causing these disruptions is increased expression of the mitochondrial calcium uniporter. Additionally, it has been shown that mice exposed to PM2.5 had lower amounts of vitamin D (VD) receptor (*VDR*) mRNA in their renal organs. To evaluate the connection between mitochondrial-dependent renal cell death, VD, and the VDR, results from HK-2 cells show that VD-VDR mainly affects mitochondrial calcium uniporter expression in response to PM2.5 exposure [189]. This regulation stabilizes MMP and limits excessive calcium input, which effectively lowers the formation of mtROS and the apoptotic response that follows [189]. Other potential interpretations regarding the excess calcium levels in mitochondria have been elaborated upon in the previous section. It was indicated that a 24 h exposure to PM2.5 results in the opening of mitochondrial permeability transition pores, which consequently leads to elevated calcium concentrations and a diminished MMP, as demonstrated in studies involving AC16 human cardiomyocyte cells [82]. In this context, the incorporation of VD presents itself as a feasible intervention to mitigate cardiomyocyte cell death and counteract cardiac hypertrophy, which could be an active area of research in the future.

### Ocular diseases

Severe retinal ischemia and associated cellular necrosis in acute retinal artery occlusion represent a catastrophic ophthalmic emergency that can precipitate permanent vision loss within a matter of hours [82], [190]. Disruption of the inner blood-retinal barrier (iBRB) is a crucial occurrence at this period. The retinal capillaries have a continuous layer of endothelium that serves as a vital barrier, protecting retinal tissue from potentially hazardous compounds in the blood [191]. Visual impairment, edema, and increased tissue osmotic pressure result from retinal vascular permeability when the iBRB is impaired [82], [192]. In conditions such as diabetic retinopathy and ischemic central retinal vein occlusion, iBRB breakdown is observed due to retinal hypoxia [193]. Limited information exists on the impact of air pollutants on the functionality of the iBRB. Following exposure to PM2.5 in an animal, experimental tests showed a major shift in retinal vascular permeability and vessel diameter, along with noticeable retinal edema and elevated inflammation [194]. The adverse effects of PM2.5 exposure on human retinal microvascular endothelial cells were shown to escalate with duration and concentration [194]. The outcomes included apoptosis, inflammatory markers, cell viability, migration, and downregulation of angiogenesis, along with heightened inflammatory indicators. Significant alterations in ferroptosis-related gene expression were also brought on by exposure to different PM2.5 concentrations, in addition to iron overload and excessive lipid oxidation. Importantly, Fer-1 may considerably reduce the harmful effects of PM2.5 on human retinal microvascular endothelial cells. This study emphasizes how exposure to PM2.5 affects vascular dilatation, retinal inflammation, and the integrity of the iBRB [194].

It has been established that PM2.5 induces ferroptosis in the lens, thereby compromising both its structure and functionality. In a study by Sheng *et al.* [9], rats were subjected to PM2.5 exposure for 3 weeks, during which they received 4 daily injections of 10 μl of a 1 mg/ml PM2.5 solution in each eye. Histological analysis employing hematoxylin and eosin staining revealed the presence of vacuoles in the equatorial region of the lens. Furthermore, assays measuring iron and GSH levels indicated that rats exposed to PM2.5 exhibited elevated concentrations of Fe^2+^ and diminished levels of GSH in their lenses. The identification of increased levels of the lipid peroxide 4-hydroxynonenal further corroborated the involvement of ferroptosis. Additionally, human lens epithelial cells exposed to PM2.5 demonstrated symptoms indicative of ferroptosis, including reduced motility and cell viability. For RNA sequencing analysis, approximately 60 human lens anterior capsule samples were collected. The results indicated that human lens anterior capsules from regions with PM2.5 concentrations at or below 30 μg/m^3^ exhibited significant differences in the expression of ferroptosis-related genes, such as GPX4 and the six-transmembrane epithelial antigen of the prostate family protein 3, when compared to those from areas with concentrations of 35 μg/m^3^ or higher. Moreover, lenses from regions characterized by elevated PM2.5 levels displayed downregulated connexin 43 expression, increased transferrin receptor expression, and heightened lipid peroxide levels [9].

Instability, hyperosmolarity, inflammation, and nerve damage are some of the symptoms of dry eye disease, a complex condition marked by an imbalance in the tear film [195]. A study indicated that PM2.5 exposure reduces mitochondrial activity, ATP levels, and cellular viability in human corneal epithelial cells (HCETs). Furthermore, exposure to PM2.5 in murine models has induced superficial punctate keratopathy and significant ocular irritation. Notably, in HCETs, there was a dose- and time-dependent increase in both cell proliferation and the formation of ROS, accompanied by a decrease in MMP and an elevation in mtROS levels. Inflammation persisted even after a brief duration of PM2.5 exposure. Nevertheless, measures including NRF2 overexpression, *NF-κB p65* knockdown, and ROS elimination have been successful in reducing the decline in ATP synthesis. These tactics significantly restored ROS, mtROS, and MMP changes while also lowering inflammation by increasing NRF2 expression and decreasing p65 levels [23].

## From bench to bedside: connecting PM2.5 toxicology and health disorders to accelerate clinical translation

As thoroughly discussed, PM2.5 has a detrimental effect on various health disorders through the implementation of RCDs. Many therapeutic agents have been evaluated in cell and animal studies to attenuate the impact of PM2.5-induced effects through impacting RCD in main health disorders **(**Table 1**)** [15], [18], [23], [24], [75], [89], [90], [92], [97], [108], [110], [116], [118], [122], [125], [152], [166], [168], [172], [194]. While various therapeutic agents have shown promise in preclinical and *in vitro* studies, the existing evidence remains limited to isolated experiments and small-scale *in vivo* tests. There is insufficient replication and no definitive human evidence to confirm their effectiveness. These early findings are preliminary and difficult to translate into widespread population strategies.

**Table 1:** The implementation of different therapeutic agents in controlling PM2.5-induced disorders through the regulation of different forms of RCD.

**Therapeutic agent**	**Cell death**	**Protective effect on disease or cell**	**Mechanism of action**	**References**
Disulfiram	Pyroptosis ↓	CVD	NLRP3, caspase-1, and GSDMD downregulation	[75]
NAC	Pyroptosis ↓	CVD	Oxidative stress control	[75]
MitoQ	Ferroptosis ↓	Cardiac fibrosis	Increase the resistance of mitochondria to lipid peroxidation	[15]
4-PBA	Autophagy and apoptosis ↓	Endothelial cells	ER stress inhibition	[89]
LBP	Autophagy ↓	HUVECs	Cellular senescence inhibition	[90]
Procyanidin	Apoptosis ↓	VSMCs	NRF2 upregulation	[92]
ZnPP	Ferroptosis ↓	Microglia Pulmonary fibrosis	HO-1 downregulation	[18], [97]
Melatonin	Ferroptosis ↓ ROS-mediated mitophagy ↓	Epilepsy Liver fibrosis Alveolar epithelial cells	Regulate cadmium toxicity NRF2 upregulation SIRT3 agonist	[23], [118], [172]
Rapamycin	Autophagy and mitophagy ↑	PD	Oxidative stress control	[116]
SVHRSP	Pyroptosis ↓ Necroptosis ↓	AD	Control the ER stress and oxidative stress LncRNA Gm16410 regulation	[108], [110]
Apyrase	Pyroptosis ↓	Bronchial epithelial cells	Antagonism of ATP	[122]
Mdivi-1	Apoptosis and mitophagy ↓	Lung epithelial cells	DRP1 inhibition	[125]
Mito-TEMPO	Ferroptosis ↓	Lung fibrosis	Mitochondrial antioxidant	[18]
SRT1720	Autophagy of Leydig cells ↑	Fertility disorder	SIRT1 activator	[166]
Deferoxamine mesylate	Ferroptosis ↓	Fertility disorder	Iron chelator	[168]
Ferrostatin-1	Ferroptosis ↓	Fertility disorder Cardiac fibrosis Ocular disease	Lipid peroxidation inhibitor Iron metabolism regulation	[24], [168], [194]
NaHS	Ferroptosis ↓	COPD	Inhibition of ferritinophagy	[152]

NLRP3. NLR family pyrin domain containing 3; GSDMD. Gasdermin D; NAC. N-acetyl-L-cysteine; MitoQ. Mitoquinone; 4-PBA. 4-phenylbutyrate; LBP. Lycium barbarum polysaccharide; ZnPP. Zinc protoporphyrin IX; HO-1. Heme oxygenase-1; SVHRSP. Scorpion venom heat-resistant synthetic peptide; SIRT. Sirtuin; HUVECs. Human umbilical vein endothelial cells; VSMCs. Vascular smooth muscle cells; NRF2. Nuclear factor erythroid 2-related factor 2; CVD. Cardiovascular disease; AD. Alzheimer’s disease; PD. Parkinson’s disease; COPD. Chronic obstructive pulmonary disease; DRP1. Dynamin-related protein 1; NaHS. Sodium hydrosulfide; RCD. Regulated cell death; ROS. Reactive oxygen species; ER. Endoplasmic reticulum; Mdivi-1. Mitochondrial division inhibitor 1; Mito-TEMPO. Mitochondria targeted antioxidant tempo

We are still in the initial stages of developing approaches to mitigate the harmful effects of PM2.5. Nevertheless, we believe that harnessing natural edible components to target oxidative stress could be a promising, safe, and practical strategy. By identifying and incorporating these natural elements as dietary supplements, we could enhance both safety and efficacy in this emerging field. A significant research gap remains concerning the alignment of preclinical studies with human clinical trials. In this context, it is essential to address several key topics. The alignment of PM2.5 exposure levels between real-world scenarios and preclinical studies is a critical factor. The world’s most populous nation, India, has reported a population-weighted mean PM2.5 exposure of 57.3 μg/m^3^ from 2000 to 2019 [196], and this value is found as 38.38 μg/m^3^ for China in 2019, which exceeds both the national and the World Health Organization standards [197]. This data provides valuable guidance for researchers to design studies with exposure doses that reflect environmental conditions, thereby enhancing the clinical relevance of their findings. For instance, numerous preclinical studies have demonstrated that exposure to PM2.5 concentrations of 35 or 40 μg/m^3^ can induce ferroptosis [9]. This suggests a hypothesis whereby controlling ferroptosis could become a priority in managing PM2.5-related health disorders in these regions.

One of the challenges in translating preclinical studies of PM2.5 exposure to clinical trials is the inconsistency in measurement indices used across various studies. For instance, researchers have employed metrics, such as milligrams per kilogram of body weight (mg/kg), micrograms per cubic meter (μg/m^3^), or milliliters (μg/ml), as indicators of exposure [9], [77], [82]. Establishing standardized measurement indices would represent a significant research gap that warrants further investigation. Another area requiring attention in PM2.5 research pertains to the evaluation of its individual components and their respective biological functions within preclinical studies. There are several characterizations of PM2.5 compositions, each underscoring the importance of considering their differences. For example, as previously mentioned, heavy metals, such as Mn and Pb, present in PM2.5 have distinct effects on tissue fibrosis [131]. Additionally, an important aspect of PM2.5 characterization involves distinguishing between water-soluble and water-insoluble components. Research has demonstrated that water-insoluble constituents exert a more detrimental influence on the regulation of RCD in multiple health disorders [73]. For instance, organic materials, which predominantly constitute insoluble components, are well-documented for their cytotoxic effects [74]. This awareness prompts researchers to focus more on targeting specific cytotoxic components within PM2.5 rather than addressing the entire spectrum of its composition.

Finally, a significant research gap in studies related to PM2.5 pertains to the lack of biomarker investigations for monitoring individuals in laboratory settings to assess the adverse effects of PM2.5 exposure. For instance, as mentioned previously, serum levels of METTL3 and STC2 have demonstrated a correlation with PM2.5 exposure [148]. This area presents a promising avenue for further research, which could potentially lead to clinical trials and strategies aimed at mitigating the detrimental effects of PM2.5. In this context, we hypothesize that evaluating markers of cellular senescence and aging may serve as effective biomarkers for PM2.5 exposure, representing a valuable focus for future investigative efforts.

## Abbreviations

α-SMA: α-smooth muscle actin

4-PBA: 4-phenylbutyrate

AD: Alzheimer’s disease

AECs: Alveolar epithelial cells

CHOP: C/EBP homologous transcription factor protein

AhR: Aryl hydrocarbon receptor

AHR: Airway hyperresponsiveness

Akt: Protein kinase B

ALKBH5: AlkB homologue 5

AMPK: Adenosine monophosphate-activated protein kinase

AR: Allergic rhinitis

ASC: Apoptosis-associated speck-like protein containing a caspase recruitment domain

ASMCs: Airway smooth muscle cells

ATF5: Activating transcription factor 5

ATG: Autophagy-related

ATP: Adenosine triphosphate

Bax: Bcl-2 associated X protein

Bcl-2: B-cell lymphoma-2

BTB: Blood-testis barrier

CDI: Chronic daily intake

CREB: Cyclic AMP response element binding protein

CK-MB: Creatine kinase MB

COPD: Chronic obstructive pulmonary disease

CVDs: Cardiovascular diseases

CYP1A1: Cytochrome P450 1A1

DRP1: Dynamin-related protein 1

EMT: Epithelial-mesenchymal transition

EOM: Extractable organic matter

ER: Endoplasmic reticulum

ERK: Extracellular signal-regulated kinase

ERS: Endoplasmic reticulum stress

FADD: Fas-associated via death domain

Fer-1: Ferrostatin-1

FTH1: Ferritin heavy chain 1

FTL: Ferritin light chain

GPX: Glutathione peroxidase

GSDMD: Gasdermin D

GSH: Glutathione

GSK-3β: Glycogen synthase kinase 3β

HCC: Hepatocellular carcinoma

HIF-1: Hypoxia-inducible factor 1

HO-1 (Hmox-1): Heme oxygenase-1

HSCs: Hepatic stellate cells

HSP60: Heat shock protein 60

HUVECs: Human umbilical vein endothelial cells

iBRB: Inner blood-retinal barrier

IFN: Interferon

IL: Interleukin

IRE-1: Inositol-requiring transmembrane kinase/endoribonuclease 1

JNK: c-Jun N-terminal kinase

KEAP1: Kelch-like ECH-associated protein 1

LBP: Lycium barbarum polysaccharide

LC3: Microtubule-associated protein 1A/1B-light chain 3

LDH: Lactate dehydrogenase

lncRNAs: Long non-coding RNAs

MAPK: Mitogen-activated protein kinase

MDA: Malondialdehyde

MerT: Myeloid-epithelial-reproductive receptor tyrosine kinase

METTL3: Methyltransferase-like 3

MitoQ: Mitoquinone

MLKL: Mixed lineage kinase domain-like protein

MMP: Mitochondrial membrane potential

Mn: Manganese

mTORC1: Mechanistic target of rapamycin complex I

mtROS**:** Mitochondrial ROS

m^6^A: N^6^-methyladenosine

NADPH: Nicotinamide adenine dinucleotide phosphate

NCOA4: Nuclear receptor coactivator 4

NLRP: NOD-like receptor family pyrin domain containing

NQO1: NAD(P)H quinone oxidoreductase 1

NRF2: Nuclear factor Erythroid 2-related factor 2

N2a: Neuro-2a

PD: Parkinson’s disease

PERK: Protein kinase RNA-like endoplasmic reticulum kinase

PI3K: Phosphoinositide 3-kinase

p-JNK: Phosphorylated c-Jun N-terminal kinase

PMLCPIR: PM2.5-induced lung cancer upregulation piRNA

PINK1: PTEN-induced kinase 1

piRNAs: P-element induced wimpy testis-interacting RNAs

PM: Particulate matter

RCD: Regulated cell death

RIPK1: Receptor-interacting protein kinase 1

ROS: Reactive oxygen species

STC2: Stanniocalcin 2

SIRT1: Silent information regulator Sirtuin 1

SIRT3: Sirtuin 3

SOD: Superoxide dismutase

solMER: Soluble MerTK

SVHRP: Scorpion venom heat-resistant peptide

SVHRSP: Scorpion Venom Heat-Resistant Synthetic Peptide

TNF-α: Tumor necrosis factor-α

TRPM2.5: Traffic-related PM2.5

ULK1: Unc-51-like autophagy activating kinase 1

UPR: Unfolded protein response

UPRmt: Mitochondrial unfolded protein response

VPS34: Vacuolar protein sorting 34

VDR: Vitamin D receptor

VSMCs: Vascular smooth muscle cells

ZBP1: Z-DNA binding protein 1

Z-DNA: Z-form DNA

ZO-1: Zonula occludens-1

## Funding

Not applicable.

## Data Availability

Not applicable.
